# Molecular Crystal Forms of Antitubercular Ethionamide with Dicarboxylic Acids: Solid-State Properties and a Combined Structural and Spectroscopic Study

**DOI:** 10.3390/pharmaceutics12090818

**Published:** 2020-08-28

**Authors:** Simone Bordignon, Paolo Cerreia Vioglio, Elena Amadio, Federica Rossi, Emanuele Priola, Dario Voinovich, Roberto Gobetto, Michele R. Chierotti

**Affiliations:** 1 Department of Chemistry and NIS Centre, University of Torino, V. Giuria 7, 10125 Torino, Italy; simone.bordignon@unito.it (S.B.); paolo.cerreiavioglio@icloud.com (P.C.V.); elena.amadio@unito.it (E.A.); fe.rossi@unito.it (F.R.); emanuele.priola@unito.it (E.P.); 2 Department of Chemical and Pharmaceutical Sciences, University of Trieste, P.le Europa 1/via L. Giorgieri 1, 34127 Trieste, Italy; vojnovic@units.it

**Keywords:** ethionamide, cocrystal, solid-state NMR, dissolution, kryptoracemate, salt cocrystal, antitubercular, API, drug, crystal engineering

## Abstract

We report on the preparation, characterization, and bioavailability properties of three new crystal forms of ethionamide, an antitubercular agent used in the treatment of drug-resistant tuberculosis. The new adducts were obtained by combining the active pharmaceutical ingredient with three dicarboxylic acids, namely glutaric, malonic and tartaric acid, in equimolar ratios. Crystal structures were obtained for all three adducts and were compared with two previously reported multicomponent systems of ethionamide with maleic and fumaric acid. The ethionamide-glutaric acid and the ethionamide-malonic acid adducts were thoroughly characterized by means of solid-state NMR (^13^C and ^15^N Cross-Polarization Magic Angle Spinning or CPMAS) to confirm the position of the carboxylic proton, and they were found to be a cocrystal and a salt, respectively; they were compared with two previously reported multicomponent systems of ethionamide with maleic and fumaric acid. Ethionamide-tartaric acid was found to be a rare example of kryptoracemic cocrystal. In vitro bioavailability enhancements up to a factor 3 compared to pure ethionamide were assessed for all obtained adducts.

## 1. Introduction

The obtainment of novel crystal forms is a well-consolidated strategy in the quest for solid molecular materials with enhanced physicochemical properties with respect to those of the pure components. The crystal engineering approach, i.e., the rational design and synthesis of new crystal forms [1], is viable for any molecule that is employed in the solid state, ranging from pigments to explosives [2], from pharmaceuticals to energy storage materials [3]. In the case of active pharmaceutical ingredients (APIs), crystal engineering has proved to be successful in modulating and improving their performances in terms of water solubility [4,5], dissolution rate [6,7], hygroscopicity [8], thermal stability [9], flow properties [10], etc. An important point of this strategy is the fact that some APIs are often doomed to obsolescence because of their poor biopharmaceutical and/or physicochemical properties [11]. Improving them represents a way to restore their value, in some cases even to reduce side effects due to a decrease in the administered doses and to extend rights on the intellectual property. This aspect gains particular importance in today’s economic and scientific context, where the R & D costs in the pharmaceutical industry increase annually [12], yet the number of approved drugs kept constant throughout 60 years [13,14]. Therefore, the revamping of old drugs represents a chance to reduce costs and also introduce new therapies [15].

The crystal engineering approach can deliver many different crystal forms, namely polymorphs, salts, solvates/hydrates and cocrystals. The latter, in particular, are more and more commonly pursued as an alternative to salts in the quest for performance enhancement of APIs, because cocrystallization offers greater opportunities than salification: (i) it is viable for molecules that do not display any ionizable moiety; (ii) the possible coformers (i.e., molecules selected to cocrystallize with the API) are more numerous than the possible counterions; (iii) cocrystallization can significantly improve the solubility of the APIs without altering their permeability. As of today, many pharmaceutical cocrystals have been successfully prepared and reported in the literature [7,16,17,18,19,20].

**Scheme 1:**
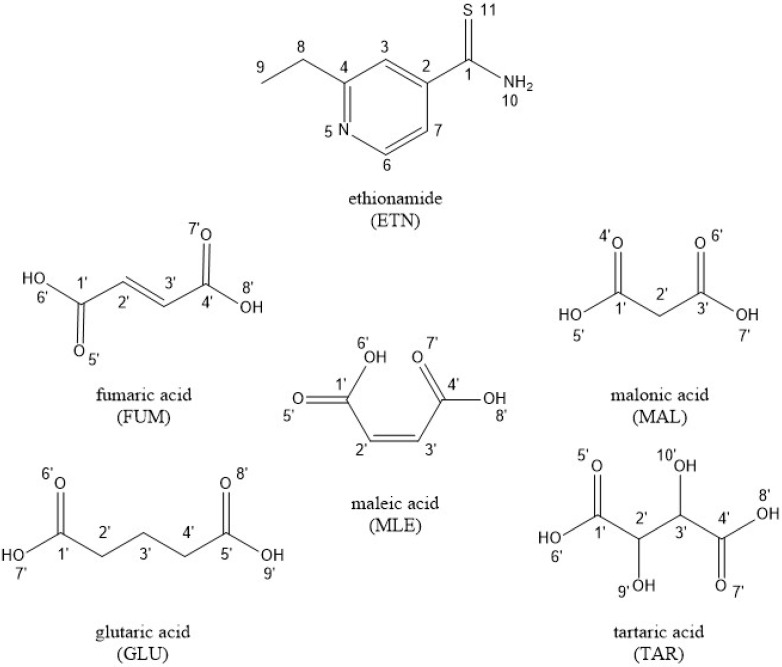
Representation of the employed molecules, with atom numbering.

This work focuses on the preparation and solid-state characterization of multicomponent crystal forms of ethionamide (2-ethylpyridine-4-carbothioamide, ETN) (Scheme 1). ETN is an anti-tubercular drug used in the treatment of drug-resistant tuberculosis; hence, it is frequently administered in association with other antibacterial agents. Indeed, multidrug- and extensively drug-resistant tuberculosis are addressed by the World Health Organization as major global issues [21]. Since ETN belongs to class II of the BCS (Biopharmaceutical Classification System), comprising of compounds with low solubility and high permeability, efforts in the improvement of the dissolution properties of ETN clearly become crucial to help the treatment of such aggressive forms of the disease. ETN crystallizes in space group *Cc* [22], without any polymorphic forms known to date. On the other hand, there are several examples in the literature of salts and cocrystals of ETN, namely ETN hydrochloride [23], hydrobromide [24], nitrate [25], oxalate [26,27], maleate [27] and saccharinate [27]; ETN·adipic acid [26], ETN·fumaric acid [26] and ETN·suberic acid [26]. Notably, in all salts reported in the literature, N5 of ETN appears to be protonated, while in all cocrystal structures of ETN with carboxylic acids, the COOH group is involved in hydrogen bond (HB) contacts with both N5 (COOH···N) and N10 (C=O···HN). Most recently, some of us managed to selectively obtain both a salt and a cocrystal for the ETN−salicylic acid system, which display remarkable in vitro bioavailability properties [16]. This further proves ETN to be a very feasible molecule to undergo the crystal engineering approach. In this paper, three new crystal forms of ETN were obtained by solution or mechanochemical techniques through the combination with glutaric (GLU), malonic (MAL) and tartaric (TAR) acid (see Scheme 1). Notably, the cocrystallization of ETN with TAR yielded a rare kryptoracemic cocrystal: to date, only one kryptoracemic cocrystal has been reported [28]. Notably, all three new crystal forms are characterized by significantly higher dissolution rates than pure ETN. 

These three novel forms were compared with two previously reported crystal forms of ETN with dicarboxylic acids fumaric (FUM) and maleic (MLE) acid [26,27], to observe similarities and differences with respect to our novel forms in terms of spectroscopic and physicochemical properties. All five forms were analyzed by single-crystal X-ray diffraction (SCXRD), while all but ETN∙TAR, which could not be reproduced as a bulk powder (see Materials and Methods section), underwent solid-state NMR (SSNMR). The latter technique was instrumental in clarifying the nature (neutral or ionic) of the obtained products, since the position of the H atoms along the HB axis was in general not clearly detected in SCXRD structures. Finally, the thermal stability and the dissolution rate were evaluated for all crystal forms (except for ETN∙TAR) by means of DSC and TGA analyses and dissolution kinetic tests (DKTs), respectively.

## 2. Materials and Methods

FUM, GLU, MLE, MAL and all solvents were purchased from Sigma-Aldrich (Milan, Italy,); ETN was purchased from Alfa Aesar (Thermo Fisher Scientific, Kendal, Germany); TAR was purchased from Schiapparelli (Carlo Erba, Cornaredo, Italy). All reagents were used without further purification.

### 2.1. Synthesis

ETN·GLU: A yellow microcrystalline powder was obtained by manually dry grinding 30 mg (0.18 mmol) of ETN and 24 mg (0.18 mmol) of GLU for 60 min. Crystals were obtained through seeding crystallization of the ground product in ethanol.

ETN·MAL: An orange microcrystalline powder was obtained by the slurry technique: 50 mg (0.3 mmol) of ETN and 31 mg (0.3 mmol) of MAL were stirred for 4 h with a few drops of ethanol. Crystals, suitable for SCXRD, were obtained through seeding crystallization of the slurried product in ethyl acetate.

ETN·TAR: Crystals were obtained through slow evaporation at room temperature of a methanol solution containing 30 mg (0.18 mmol) of ETN and 27 mg (0.18 mmol) of TAR. Despite many attempts, ETN·TAR could not be reproduced in pure form to undergo further analyses.

ETN·FUM: Crystals were obtained through slow evaporation at room temperature of a methanol solution containing 30 mg (0.18 mmol) of ETN and 21 mg (0.18 mmol) of FUM.

ETN·MLE: An orange microcrystalline powder was obtained by manually dry grinding 30 mg (0.18 mmol) of ETN and 21 mg (0.18 mmol) of MLE for 30 min. Crystals, suitable for SCXRD, were obtained through slow evaporation at room temperature of an acetone solution containing 15 mg (0.09 mmol) of ETN and 10.5 mg (0.09 mmol) of MLE. 

### 2.2. Screening Techniques

#### Raman Spectroscopy

Raman spectra were registered with a Bruker Vertex 70 instrument (Bruker, Billerica, MA, USA), equipped with a RAM II module. An excitation source at 1064 nm was used, with a laser power between 10 and 50 mW and a number of scans between 80 and 500, depending on the analyzed sample, with a resolution of 4 cm^−1^. The employed spectral range is comprised between 50 and 4500 cm^−1^, using a CaF_2_ beam splitter. Raman spectra are not discussed as they were used only for screening purposes, but they are reported in Figures S1–S4.

### 2.3. Characterization Techniques

#### 2.3.1. X-Ray Diffraction (SCXRD and PXRD)

Single crystals of ETN·GLU, ETN·MAL and ETN·TAR were analyzed with a Gemini R Ultra diffractometer (Rigaku Oxford Diffraction, Abingdon, Oxfordshire, UK) operating at 293(2) K, using a Mo Kα source (λ = 0.71073 Å). Data collection and reduction were performed using the CrysAlisPro software (Rigaku Oxford Diffraction, Abingdon, Oxfordshire, UK). The crystal structure was solved by direct methods and refined with the full matrix least-squares technique on F2 using the SHELXS-97 and SHELXL-97 programs (Structural Chemistry Department at the University of Göttingen, Germany). All non-hydrogen atoms were refined anisotropically; hydrogen atoms bonded to unambiguous sites were placed in geometrical positions and refined using the riding model. Hydrogen atoms between pyridinic nitrogen and carboxylic oxygen sites of nearby molecules have been detected in the Fourier maps, and their position has been further confirmed through SSNMR. See Table 1 for the crystal data and structure refinement parameters for ETN·GLU, ETN·MAL and ETN·TAR, and Tables S2–S7 for the measured crystallographic distances and angles (refer to Scheme 1 for atom numbering).

ETN·TAR presents a kryptoracemic structure, and the absence of an inversion center (although quite certain from the near 0 Flack parameter) [29] or other second-type symmetry elements has been checked by pseudosymmetry search using the PSEUDO program [30] of Bilbao Crystallographic Server, and no centrosymmetric supergroup compatible with the experimental atomic positions has been found. 

Powder diffractograms were obtained on the same Gemini R Ultra diffractometer (Rigaku Oxford Diffraction, Abingdon, Oxfordshire, UK), equipped with an X-ray source using Cu Kα radiation (λ = 1.54 Å). Data were collected and processed through the CrysAlisPro software.

CCDC accession codes 2019883, 2019884 and 2019885 contain the supplementary crystallographic data for ETN·MAL, ETN·GLU and ETN·TAR, respectively. These data can be obtained free of charge via www.ccdc.cam.ac.uk/data_request/cif, or by e-mailing data_request@ccdc.cam.ac.uk, or by contacting The Cambridge Crystallographic Data Centre, 12 Union Road, Cambridge CB2 1EZ, UK; fax: +44-1223-336033.

#### 2.3.2. Solid-State NMR Measurements

^13^C CPMAS and ^15^N CPMAS SSNMR spectra were collected on a Bruker Avance II 400 Ultra Shield instrument (Bruker, Billerica, MA, USA), working at 400.23, 100.63, and 40.56 MHz for ^1^H, ^13^C and ^15^N, respectively. Samples were packed in cylindrical zirconia rotors (4 mm o.d., Bruker, Billerica, MA, USA), with a sample volume of 80 μL. ^13^C and ^15^N spectra were acquired at room temperature with a rotation frequency of 12 and 9 kHz, respectively. All ^13^C and ^15^N experiments employed the RAMP-CP pulse sequence (^1^H 90° pulse = 3.6 μs; contact time = 4 ms) with the TPPM ^1^H decoupling (rf field = 69.4 kHz) during the acquisition period. Detailed acquisition parameters (number of scans, relaxation delays, contact times) may be found in Table S7. All employed relaxation delay values were optimized on each sample by means of ^1^H saturation recovery experiments and obtained by multiplying the measured T_1_^1^H values by 1.27, to ensure full relaxation. ^13^C and ^15^N chemical shift scales were referenced with the resonance of glycine (^13^C methylene signal at 43.5 ppm), (NH_4_)_2_SO_4_ (^15^N signal at 24.6 ppm with respect to NH_3_), respectively, as external standards.

#### 2.3.3. Thermal Analyses

TGA measurements (TA Instruments, New Castle, UK) were performed over a temperature range of 30–350 °C under a 50 mL·min^−1^ N_2_ flow, on a Q600 SDT TA instrument equipped with a DSC heat flow analyzer. Samples (5–10 mg of weight) were placed into the furnace inside alumina crucibles and heated with a ramp of 10°C·min^−1^. DSC curves were collected on a DSC Q200 TA Instrument (TA Instruments, New Castle, UK). Samples were accurately weighed (5–10 mg) and put into sealed aluminum pans. Calibration for temperature and heat flow was performed using high purity standards of n-decane, benzene and indium. All measurements were performed in a 30–350 °C temperature range, with heating rates of 10 °C·min^−1^. 

#### 2.3.4. Dissolution Kinetic Tests (DKTs)

DKTs were carried out in phosphate buffer (pH = 7.4). For each measurement, 4 mg of either ETN or its adducts were added to 100 mL of the thermostatically controlled (at 37 °C) dissolution medium. Dissolution parameters were evaluated for 60 min. The solution was kept homogeneous by continued stirring at 100 rpm, and concentrations were measured using an optical fiber system (HELLMA, Milan, Italy) linked to a spectrophotometer. UV measurements (ZEISS, Wetzlar, Germany) were performed at the maximum absorption wavelength of ETN, namely 288 nm. A calibration curve (Figure S5) was obtained with five diluted ETN solutions in phosphate buffer (the concentrations used were the following: 8, 10, 16, 20 and 40 mg/L), while pure phosphate buffer was used as the blank. 

## 3. Results and Discussion

Three novel crystal forms were obtained by means of solution or mechanochemical techniques. These are a salt of ETN with malonic acid (ETN·MAL), a cocrystal between ETN and glutaric acid (ETN·GLU) and a salt cocrystal of ETN with tartaric acid (ETN·TAR). Two crystal forms of ETN were reproduced from the literature, namely a salt of ETN with maleic acid (ETN·MLE) [27] and a cocrystal between ETN and fumaric acid (ETN·FUM) [26]. Table 2 summarizes the techniques used for preparing the new crystal forms and the outcome, in terms of stoichiometry and ionization state. 

The crystal structures of all the adducts were obtained through SCXRD. Moreover, for each adduct, except ETN·TAR, which, despite several attempts, could not be reproduced to undergo further analysis, the XRD powder patterns calculated from crystal structures were compared to the experimental powder diffractograms obtained from bulk powders to confirm that the selected crystals were representative of the whole product (see Figures S6–S9).

### 3.1. SCXRD

#### 3.1.1. ETN·GLU

**Figure 1:**
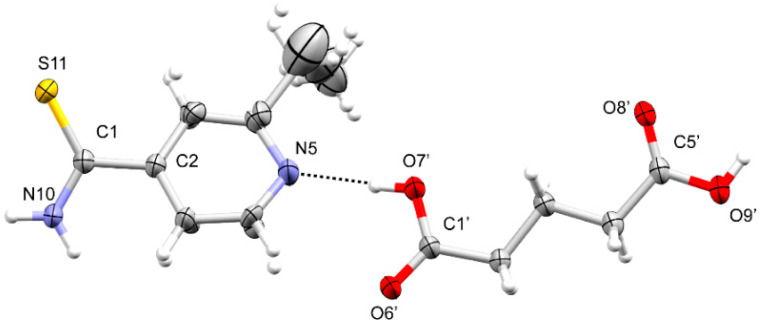
Asymmetric unit of ETN·GLU.

ETN·GLU crystallizes in the centrosymmetric triclinic space group P*1*. The asymmetric unit (Figure 1) includes one molecule of ETN and one of GLU, which interact with each other through a HB between pyridinic N5 and one of the carboxylic moieties (d N5–O7′ = 2.654 (4) Å). The neutral nature of the adduct is confirmed by the C–O (d C1′–O6′ = 1.203 (3) Å, d C1′–O7′ = 1.295 (3) Å, d C5′–O9′ = 1.315 (3) Å, d C5′–O8′ = 1.217 (2) Å) distances, consistent with the distribution of distances obtained by the CSD results for neutral COOH groups; this is supported by the ^15^N CPMAS NMR spectrum as well (see the SSNMR paragraph). Notably, ETN displays rotational disorder around the axis formed by atoms C1, C2, N5 and O7′, as represented by the doubling of the thermal ellipsoids (Figure 1).

**Figure 2:**
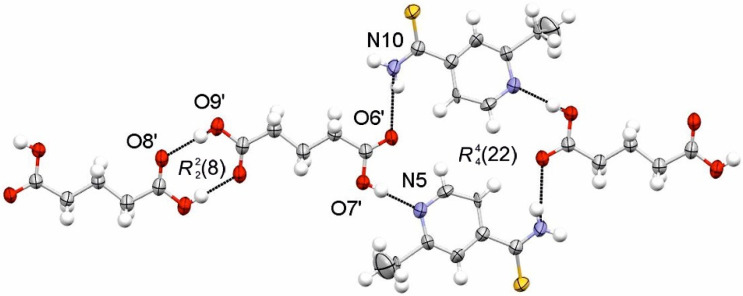
Hydrogen bond (HB) pattern of ETN·GLU, highlighting the centrosymmetric $R_{2}^{2}$(8) dimer and the $R_{4}^{4}$ (22) cyclic motif.

The HB pattern (Figure 2) is characterized by the presence of a centrosymmetric $R_{2}^{2}$(8) dimer, formed between the carboxylic groups of two GLU molecules (d O8′–O9′ = 2.667 (4) Å). Additionally, the COOH groups of GLU molecules, which are not involved in the formation of the dimers, interact with the NH_2_ groups (d N10–O6′ = 2.932 (4) Å) and with the pyridinic N5 (see above) of two different ETN molecules. The result is the formation of $R_{4}^{4}$(22) cyclic motifs, as visible in Figure 2. 

#### 3.1.2. ETN·MAL

**Figure 3:**
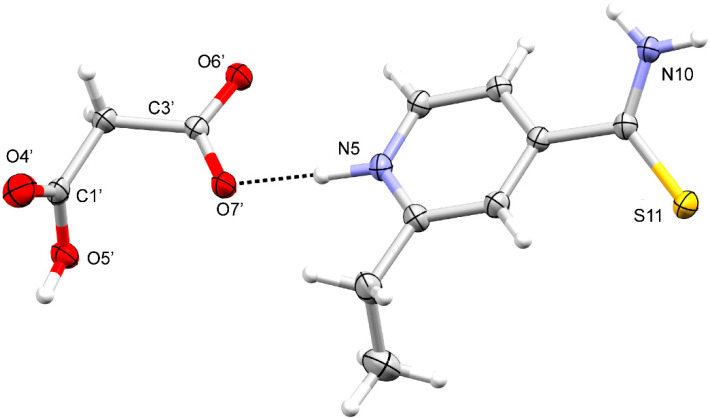
Asymmetric unit of ETN-MAL.

ETN·MAL crystallizes in the centrosymmetric triclinic space group P*1*. The asymmetric unit (Figure 3) contains one ETN molecule, protonated on N5 (d N5–O7′ = 2.657(4) Å) and one MAL molecule, characterized by a carboxylate group (d C3′–O7′ = 1.264(3) Å and C3′–O6′ = 1.234(3) Å) and a carboxylic moiety (d C1′–O4′ = 1.196 (3) Å and d C1′–O5′ = 1.329 Å). 

**Figure 4:**
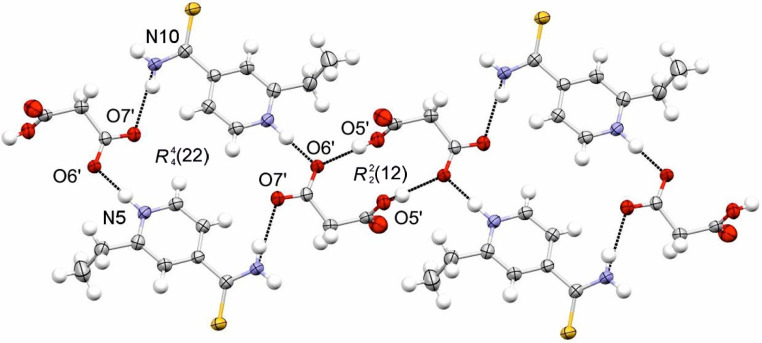
HB pattern of ETN·MAL, highlighting the centrosymmetric $R_{2}^{2}$(12) dimer and the $R_{4}^{4}$ (22) cyclic motif.

The HB pattern (Figure 4) is characterized by the presence of two O–H···O^−^ interactions between two molecules of MAL through their COOH and COO^−^ groups, which form a centrosymmetric $R_{2}^{2}$(12) dimer (d O6′–O5′ = 2.653 (3) Å). The carboxylate group also interacts with the N5 and N10 centers of ETN molecules forming N5^+^–H···O^−^ and N10–H···O contacts (d N5–O6′ = 2.657 (4) Å; d N10–O7′ = 2.844 (4) Å), leading to the formation of the $R_{4}^{4}$(22) cyclic motifs already observed in ETN·GLU.

#### 3.1.3. ETN·TAR

**Figure 5:**
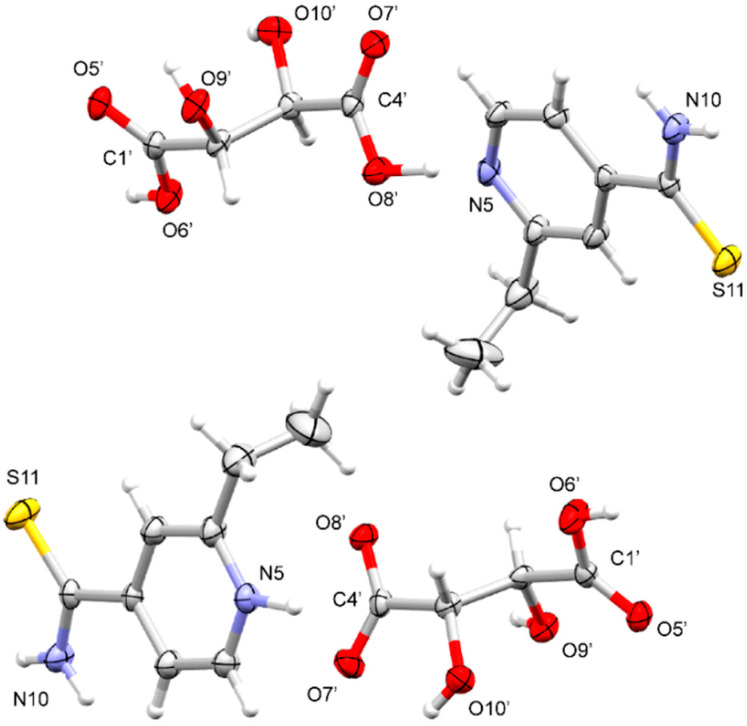
Asymmetric unit of ETN·TAR.

ETN·TAR crystallizes in a monoclinic non-centrosymmetric *P*2_1_ space group. It is a kryptoracemate, i.e., a compound that crystallizes in a non-centrosymmetric space group containing only symmetry elements of the first type (Sohnke group), despite containing both the enantiomers of a molecule in the same lattice [31]. This phenomenon is still rarely detected in both organic [31] and organometallic [32] crystals (0.1% of all the structure reported in the CSD database), although some attempt to rationally develop some functional material based on this peculiarity has been considered [33,34]. Its explanation is deeply debated, although it is clearly related to the existence of high Z’ structures and pseudosymmetry [35]. However, the existence of the first kryptoracemic cocrystal has been reported only in 2016 [28], making our result quite peculiar. In the asymmetric unit (Figure 5), four molecules are present: two ETN molecules and two TAR molecules (both enantiomers). 

**Figure 6:**
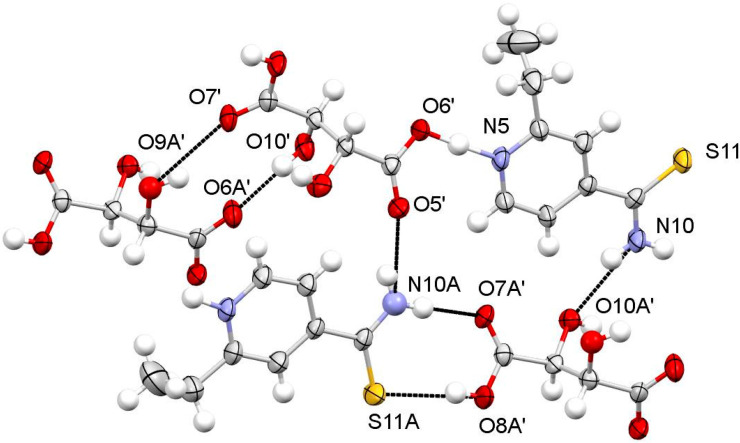
HB interactions between chains in ETN·TAR.

The two crystallographically independent TAR molecules significantly differ in the C–O distances of the carboxylic moieties. Indeed, for one molecule (top in Figure 5), d C4′–O7′ = 1.215 (2) Å, d C4′–O8′ = 1.291 (2) Å, d C1′–O6′ = 1.325 (2) Å and d C1′–O5′ = 1.211 (2) Å, while, for the other one (bottom in Figure 5), d C1′–O5′ = 1.188 (2) Å, d C1′–O6′ = 1.327 (2) Å, d C4′–O7′ = 1.228 (2) Å and d C4′–O8′ = 1.260 (2) Å. This introduces a degree of uncertainty in the position of the H atoms along the HB axes, which makes it complicated to assess the neutral or ionic nature of the adduct. Unfortunately, it was not possible to confirm it by means of SSNMR. As far as the X-ray analysis is concerned, the structure can be defined as a salt cocrystal as ETN is present is in both its neutral and ionic forms. In the HB pattern (Figure 6), chains of alternated ETN and TAR molecules are present. They are linked by HB N5^+^–H···O^−^ interactions and $R_{2}^{2}$(8) motifs involving the thioamidic (ETN) and the carboxylic (TAR) groups. Since TAR is present in both its enantiomeric forms, the two strands differ in terms of chirality, making the distances not equivalent. The bottom molecule in Figure 5 displays the following distances: d N5–O8′ = 2.571 (4) Å, d S11–O6′ = 3.144 (2) Å and d N10–O5′ = 3.025 (4) Å. The top molecule in Figure 5 presents the following distances: d N5–O8′ = 2.549 (4) Å, d S11–O6′ = 3.088 (2) Å and d N10–O5′ = 2.960 (4) Å.

The chains interact with a complex pattern of HBs involving all OH and carboxylic groups of TAR and the thioamidic group of ETN. 

**Figure 7:**
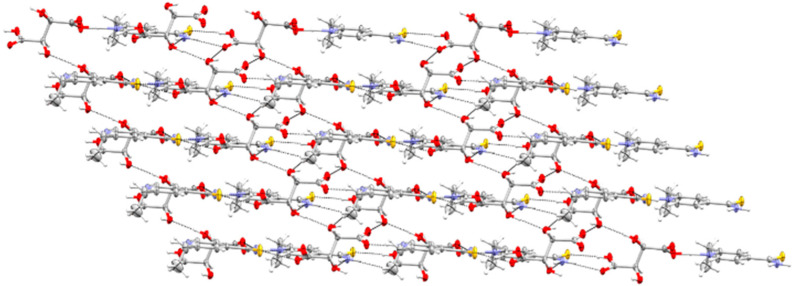
Links between chains through HBs in ETN·TAR.

The presence of both enantiomers of TAR distinguishes the chains in the disposition of the OH groups, generating a double layer (Figure 7).

The structures of ETN·FUM and ETN·MLE are already discussed in [26] and [27]. For the sake of clarity, the asymmetric units and the HB networks are reported in Figures S10–S13.

### 3.2. SSNMR

**Figure 8:**
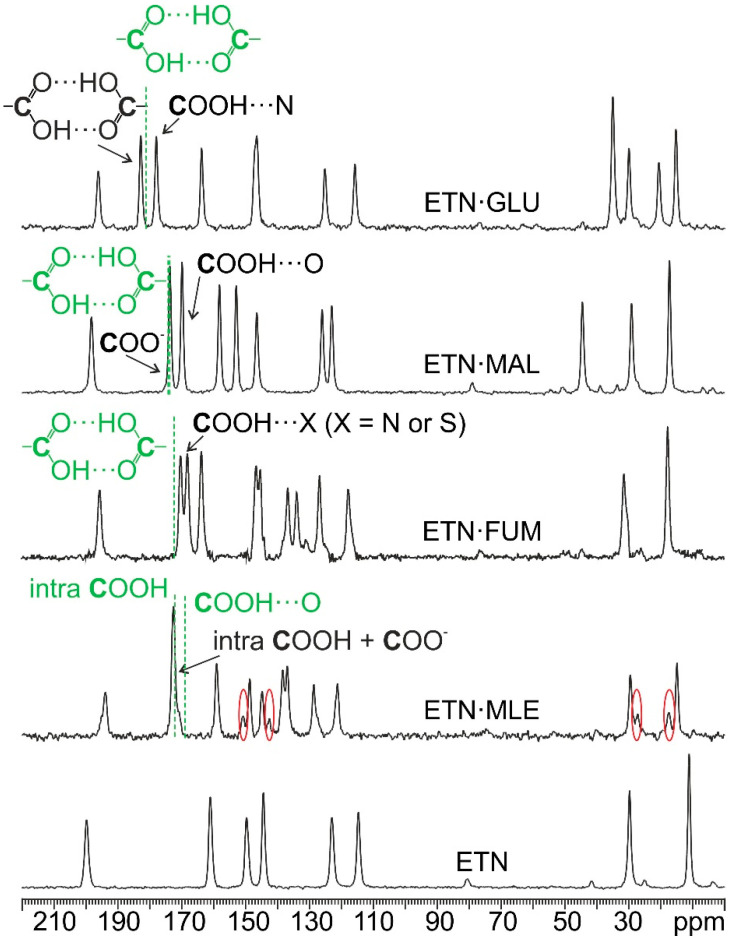
^13^C CPMAS spectra of the analyzed compounds. Dashed green lines and green writings refer to carboxylic signals of the pure acids. Red ovals highlight extra peaks due to crystalline disorder.

**Figure 9:**
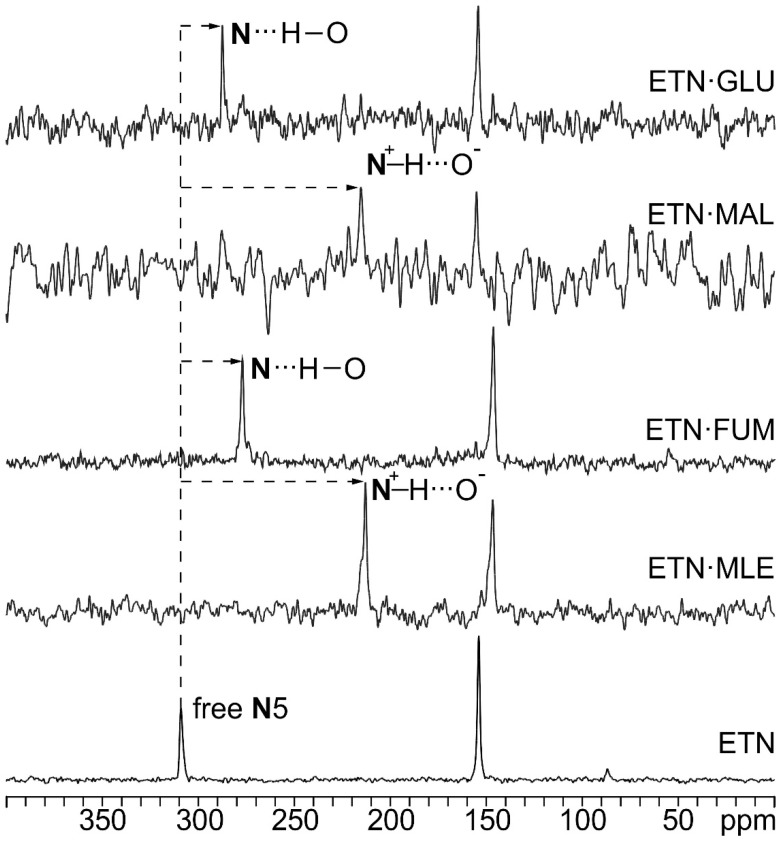
^15^N CPMAS spectra of the analyzed compounds. Dashed lines highlight the chemical shift of N5 in pure ETN.

SSNMR was useful to verify the neutral or ionic nature of all adducts, except ETN·TAR, strengthening the X-ray evidence [36,37,38,39]. Indeed, the position of the H atoms along the HB axis was not always clearly detected from X-ray analyses. Through 1D ^13^C CPMAS (Figure 8) and ^15^N CPMAS (Figure 9) experiments, the SSNMR analysis focused on the ^13^C resonances of the carboxylic groups of the acids and the ^15^N signals of N5 (pyridinic) and N10 (thioamidic) of ETN. Indeed, these chemical shifts are very sensitive to the protonation state of the corresponding moieties [37]. All ^13^C and ^15^N chemical shifts with their relative assignments are reported in Table 3. 

^13^C CPMAS spectra offer the chance to assess the involvement of the carboxylic groups of the coformers in deprotonation or HB contacts. The spectrum of ETN·GLU exhibits two carboxylic resonances at 182.7 and 177.8 ppm. This can be explained by the variation in the network of interactions engaging the two COOH moieties. The former is assigned to a COOH group (182.7 ppm) forming a homodimeric $R_{2}^{2}$(8) synthon with neighboring GLU molecules, as also observed in pure GLU (181.4 ppm) [40]. This translates into high-frequency chemical shifts for carboxylic groups, comparable to those typical of carboxylate moieties [37]. In ETN·GLU, the second resonance (177.8 ppm) is typical of neutral COOH groups, in this case engaging in a COOH···N HB. This nicely agrees with X-ray data as in the case of ETN·MAL. As a matter of fact, pure MAL displays carboxylic homodimers (δ = 174.3/174.8 ppm) [41], while, in ETN·MAL, we attribute the signal at 173.6 ppm to a COO^−^ group and the remaining peak (169.8 ppm) to a COOH moiety involved in a $R_{2}^{2}$(12) dimeric COOH···O HB. Homodimers are also characteristic of pure FUM [42] (172.3 ppm); on the contrary, in ETN·FUM [26], both COOH groups stay protonated and are no longer involved in homodimeric interactions, which explains the low-frequency shift of their resonances (170.0 and 167.9 ppm). Pure MLE represents an exception, since it does not exhibit homodimeric synthons [43], which justifies the relatively low chemical shift of one of the COOH groups (169.2 ppm); the other COOH is involved in a COOH···O intramolecular interaction, bringing its chemical shift up to 172.7 ppm. In ETN·MLE [27], a single resonance can be observed, at 172.5 ppm. This can be traced back to the high symmetry of the hydrogenmaleate group, which leads the two carboxylic groups to be very similar (d C4′–O8′= 1.272 Å; d C4′–O7′= 1.241 Å and d C1′–O6′= 1.285 Å; d C1′–O5′= 1.233 Å, atom numbering in Figure S12) despite the deprotonation of one of them. Notably, the spectrum of the salt exhibits extra peaks in the aliphatic and aromatic regions with lower intensity, specifically those centered at about 17, 27, 142 and 151 ppm (highlighted with red ovals in Figure 8). These are due to disorder associated to the ethyl and pyridyl groups, as also observed in the crystal structure (see Figure S12). 

In SSNMR, the ^15^N chemical shift is recognized as being particularly sensitive and highly reliable on the position of neighboring protons [37]. As indicated by the drastic low-frequency shift (Δδ > 80 ppm) of the N5 signal of ETN from 308.9 ppm (pure ETN) to 215.4 ppm (ETN·MAL) and 212.7 ppm (ETN·MLE), the two adducts are confirmed to be salts, in fair agreement with X-ray measurements [27]; in ETN·FUM and ETN·GLU, the N5 signal shifts to lower frequencies as well (276.9 and 287.4 ppm, respectively), but the variation is lower than for ETN·MAL or ETN·MLE (Δδ ≈ 32 and 21 ppm), and it is consistent with the formation of a HB involving N5 rather than a proper proton transfer [26]. This indicates the neutral nature of ETN·FUM and ETN·GLU, which are to be considered cocrystals, confirming the X-ray findings. 

### 3.3. Thermal Analyses

Thermal analyses were run to evaluate the thermal behavior of the adducts with respect to pure ETN, which melts at 165.6 °C. The corresponding curves are reported in Figures S14–S21. Table 4 reports all the obtained values. In all cases, endothermic DSC peaks, corresponding to lower melting points than for pure ETN, are observed. This behavior is recurrent for ETN as all the adducts reported in literature are characterized by lower melting points [16,25,26,27].

### 3.4. Dissolution Kinetic Tests

**Figure 10:**
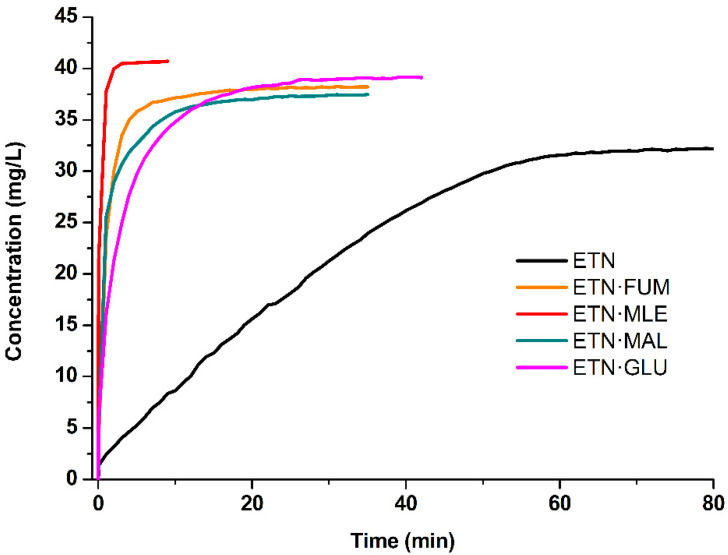
Dissolution profiles of pure ETN and all obtained adducts.

The dissolution rate for all obtained adducts, except for ETN·TAR, was evaluated in order to assess its variation with respect to pure ETN. Dissolution tests were already performed at pH = 1.2 for ETN·FUM [23] and ETN·GLU [23]. To the best of the authors’ knowledge, this is the first time they are conducted at physiological pH values (7.4). Concentrations (mg/L) were plotted against time (min), as shown in Figure 10. The dissolution rate of ETN in ETN·MLE is the highest among the obtained adducts. Nonetheless, a significant improvement in the dissolution rate of ETN is observed for all of them. The ratios between the Area Under the Curve (AUC) values of each adduct and pure ETN are reported in Table 5. This parameter allows one to assess the increase in the in vitro bioavailability of ETN in the crystal forms [44]. In all cases, a remarkable increase from two up to eight times is observed.

## 4. Conclusions

ETN proved promising to engineer new crystal forms with enhanced physicochemical properties. The presence in its molecular structure of a thioamidic moiety and of a heterocyclic N atom makes it easy to salify or cocrystallize with dicarboxylic acids. Three new crystal forms were obtained—namely, a salt (ETN-MAL), a cocrystal (ETN-GLU) and a salt-cocrystal (ETN-TAR). As in all cases reported in the literature, in ETN-MAL and ETN-TAR, N5 is protonated, while, in ETN-GLU and ETN-TAR, COOH···N and C=O···HN contacts are present. The salt cocrystal with TAR presents the rare characteristic to be a kryptoracemic cocrystal, a racemate that crystallizes into a Sohnke group; this behavior can be attributed to the concomitant presence of both the enantiomers in the asymmetric unit with some degree of distortion between each other, preserving their generation through an inversion center, a mirror plane or a glide.

The solid-state characterization of all the adducts was performed by SCXRD analyses and supported by ^13^C and ^15^N CPMAS SSNMR experiments. The latter are particularly informative, since they provide unambiguous results. These made it possible to assess the purity, the degree of crystallinity and the ionic/neutral nature, clarifying the exact position of protons, which were often uncertain in the obtained X-ray structures.

As for their physicochemical properties, all analyzed adducts show lower melting points than pure ETN. In this sense, by also comparing literature data (more than 10 adducts), we can affirm that cocrystallization systematically decreases its melting point. The dissolution profile for each analyzed adduct was evaluated. Their dissolution rates all proved significantly higher than for the pure API. In particular, ETN·MLE stands out as eight times more bioavailable (in vitro) than pure ETN.

## Figures and Tables

**Table 1:** Crystal data and structure refinement parameters for ETN·GLU, ETN·MAL and ETN·TAR.

**ETN·GLU**	
Empirical formula	C_13_H_18_N_2_O_4_S
Formula weight	298.35
Temperature/K	293(2)
Crystal system	triclinic
Space group	P-1
a/Å	5.3818(3)
b/Å	11.4336(6)
c/Å	13.2901(9)
α/°	78.196(5)
β/°	80.618(5)
γ/°	77.435(5)
Volume/Å^3^	775.28(8)
Z	2
ρ_calc_g/cm^3^	1.278
μ/mm^−1^	0.222
F(000)	316.0
Crystal size/mm^3^	0.50 × 0.22 × 0.21
Radiation	Mo Kα (λ = 0.71073)
2Θ range for data collection/°	6.74 to 50.06
Index ranges	−6 ≤ h ≤ 6, −13 ≤ k ≤ 13, −14 ≤ l ≤ 15
Reflections collected	5457
Independent reflections	2739 (R_int_ = 0.0228, R_sigma_ = 0.0543)
Data/restraints/parameters	2739/25/238
Goodness-of-fit on F^2^	1.043
Final R indexes [I >= 2σ (I)]	R_1_ = 0.0448, wR_2_ = 0.1131
Final R indexes [all data]	R_1_ = 0.0593, wR_2_ = 0.1236
Largest diff. peak/hole/e Å^−3^	0.18/−0.18
**ETN·MAL**	
Empirical formula	C_11_H_14_N_2_O_4_S
Formula weight	270.30
Temperature/K	293(2)
Crystal system	triclinic
Space group	P-1
a/Å	7.0008(12)
b/Å	8.8110(13)
c/Å	11.1507(13)
α/°	88.116(11)
β/°	77.358(12)
γ/°	68.930(15)
Volume/Å^3^	625.48(16)
Z	2
ρ_calc_g/cm^3^	1.435
μ/mm^−1^	0.267
F(000)	284.0
Crystal size/mm^3^	0.256 × 0.210 × 0.203
Radiation	Mo Kα (λ = 0.71073)
2Θ range for data collection/°	6.66 to 50.04
Index ranges	−8 ≤ h ≤ 7, −10 ≤ k ≤ 10, −13 ≤ l ≤ 10
Reflections collected	4108
Independent reflections	2204 (R_int_ = 0.0343, R_sigma_ = 0.0743)
Data/restraints/parameters	2204/0/170
Goodness-of-fit on F^2^	1.038
Final R indexes [I >= 2σ (I)]	R_1_ = 0.0422, wR_2_ = 0.0974
Final R indexes [all data]	R_1_ = 0.0551, wR_2_ = 0.1065
Largest diff. peak/hole/e Å^−3^	0.19/−0.23
**ETN·TAR**	
Empirical formula	C_24_H_32_N_4_O_12_S_2_
Formula weight	632.66
Temperature/K	293(2)
Crystal system	monoclinic
Space group	P2_1_
a/Å	4.8561(3)
b/Å	24.2003(13)
c/Å	12.1304(6)
α/°	90.00
β/°	92.671(5)
γ/°	90.00
Volume/Å^3^	1424.00(14)
Z	2
ρ_calc_g/cm^3^	1.475
μ/mm^−1^	0.257
F(000)	664.0
Crystal size/mm^3^	0.42 × 0.41 × 0.21
Radiation	Mo Kα (λ = 0.71073)
2Θ range for data collection/°	6.72 to 50.04
Index ranges	−4 ≤ h ≤ 5, −28 ≤ k ≤ 26, −14 ≤ l ≤ 14
Reflections collected	11036
Independent reflections	4925 (R_int_ = 0.0420, R_sigma_ = 0.0)
Data/restraints/parameters	4925/1/395
Goodness-of-fit on F^2^	1.036
Final R indexes [I >=2σ (I)]	R_1_ = 0.0475, wR_2_ = 0.0852
Final R indexes [all data]	R_1_ = 0.0659, wR_2_ = 0.0918
Largest diff. peak/hole/e Å^−3^	0.23/−0.22
Flack parameter	0.07(7)

**Table 2:** Summary of the employed techniques used for the solid-state preparations, with final stoichiometry and ionization state for all obtained adducts.

Crystal Form	Preparation Technique	Final Stoichiometry	Outcome
ETN∙GLU	Dry grinding	1:1	Cocrystal
ETN∙MAL	Slurry in ethanol	1:1	Salt
ETN∙TAR	Slow evaporation from methanol	1:1	Salt cocrystal

**Table 3:** ^13^C and ^15^N SSNMR chemical shift values for all analyzed powders. The atom numbering is referred to as Scheme 1.

Atom	ETN (ppm)	MAL (ppm)	GLU (ppm)	MLE (ppm)	FUM (ppm)	ETN·MAL (ppm)	ETN·GLU (ppm)	ETN·MLE (ppm)	ETN·FUM (ppm)
**^13^C**									
1	199.7					198.1	196.0	193.8	195.3
2	149.6					152.8	147.0	148.6	146.4
3	122.9					125.9	125.1	128.5	126.6
4	161.0					158.0	163.6	159.0	163.5
6	144.4					146.4	146.4	144.7	144.9
7	114.6					122.9	115.7	121.1	117.6
8	29.7					29.1	29.9	29.5	31.3
9	11.1					17.2	15.2	14.9	17.6
1’		174.3	181.4	169.2	172.3	169.8	177.8	172.5	167.9
2’		40.6	33.8	133.1	136.2	44.5	34.9	136.8	133.7
3’		174.8	18.7	140.0	136.2	173.6	20.6	138.2	136.4
4’			33.8	172.7	172.3		34.9	172.5	170.0
5’			181.4				182.7		
**^15^N**									
5	308.9					215.4	287.4	212.7	276.9
10	153.8					154.6	153.8	146.2	146.2

**Table 4:** TGA onset points and DSC signal max values for pure ETN and all obtained adducts. See Figures S14–S21.

TGA Onset Points (°C)		DSC Signal Max (°C)	
ETN	195.1	ETN	165.6
ETN·FUM	183.6	ETN·FUM	148.9
ETN·GLU	191.0	ETN·GLU	105.6
ETN·MLE	162.8	ETN·MLE	142.3
ETN·MAL	165.2	ETN·MAL	95.2

**Table 5:** Normalized Area Under the Curve (AUC) values obtained by the dissolution curves of the obtained ETN adducts.

	AUC/AUC_ETN_
ETN·FUM	2.7
ETN·GLU	2.3
ETN·MLE	7.8
ETN·MAL	2.6

## Supplementary Materials

The following are available online at https://www.mdpi.com/1999-4923/12/9/818/s1, Figures S1–S4: Raman spectra, Tables S1–S6: Crystallographic distances and angles for the crystal structures of ETN·MAL, ETN·GLU and ETN·TAR; Table S7: SSNMR acquisition parameters; Figure S5: DKT calibration curve; Figures S6–S9: PXRD patterns; Figures S10–S13: Asymmetric units and hydrogen bond patterns for ETN·MLE and ETN·FUM; Figures S14–S17: TGA curves; Figures S18–S21: DSC thermograms; The CIF and the checkCIF output files are included in the Supplementary Materials.
