# Supplementary material for: Molecular Crystal Forms of Antitubercular Ethionamide with Dicarboxylic Acids: Solid-State Properties and a Combined Structural and Spectroscopic Study
Source: Pharmaceutics. 2020 Aug 28;12(9):818. doi: 10.3390/pharmaceutics12090818 (PMC7559828; doi:10.3390/pharmaceutics12090818)

checkCIF/PLATON report

Structure factors have been supplied for datablock(s) preex1

THIS REPORT IS FOR GUIDANCE ONLY. IF USED AS PART OF A REVIEW PROCEDURE FOR PUBLICATION, IT SHOULD NOT REPLACE THE EXPERTISE OF AN EXPERIENCED CRYSTALLOGRAPHIC REFEREE.

No syntax errors found. CIF dictionary Interpreting this report

Datablock: preex1

```
Bond precision:  C-C = 0.0031 A          Wavelength=0.71073

Cell:          a=7.0008(12)       b=8.8110(13)     c=11.1507(13)
               alpha=88.116(11)   beta=77.358(12)  gamma=68.930(15)
Temperature:   293 K

                 Calculated                 Reported
Volume           625.48(18)                 625.48(16)
Space group      P -1                       P-1
Hall group       -P 1                       ?
Moiety formula   C8 H11 N2 S, C3 H3 O4      ?
Sum formula      C11 H14 N2 O4 S            C11 H14 N2 O4 S
Mr               270.30                     270.30
Dx,g cm-3        1.435                      1.435
Z                2                          2
Mu (mm-1)        0.267                      0.267
F000             284.0                      284.0
F000’            284.39
h,k,lmax         8,10,13                    8,10,13
Nref             2210                       2204
Tmin,Tmax        0.935,0.947
Tmin’            0.934

Correction method= Not given

Data completeness= 0.997          Theta(max)= 25.020

R(reflections)= 0.0422( 1775)     wR2(reflections)= 0.1065( 2204)

S = 1.038                 Npar= 170
```

The following ALERTS were generated. Each ALERT has the format
test-name_ALERT_alert-type_alert-level.
Click on the hyperlinks for more details of the test.

Alert level C

```
PLAT910_ALERT_3_C Missing # of FCF Reflection(s) Below Theta(Min)           6 Note
```

Alert level G

```
PLAT005_ALERT_5_G No Embedded Refinement Details found  in the CIF     Please Do !
PLAT007_ALERT_5_G Number of Unrefined Donor-H Atoms ..............          3 Report
PLAT152_ALERT_1_G The Supplied and Calc. Volume s.u. Differ by ...          2 Units
PLAT199_ALERT_1_G Reported _cell_measurement_temperature ..... (K)        293 Check
PLAT200_ALERT_1_G Reported   _diffrn_ambient_temperature ..... (K)        293 Check
PLAT899_ALERT_4_G SHELXL97   is Deprecated and Succeeded by SHELXL       2014 Note
PLAT909_ALERT_3_G Percentage of Observed Data at Theta(Max) Still          64 % Note
PLAT978_ALERT_2_G Number C-C Bonds with Positive Residual Density.          2 Note
```

```
   0 ALERT level A = Most likely a serious problem - resolve or explain
   0 ALERT level B = A potentially serious problem, consider carefully
   1 ALERT level C = Check. Ensure it is not caused by an omission or oversight
   8 ALERT level G = General information/check it is not something unexpected

   3 ALERT type 1 CIF construction/syntax error, inconsistent or missing data
   1 ALERT type 2 Indicator that the structure model may be wrong or deficient
   2 ALERT type 3 Indicator that the structure quality may be low
   1 ALERT type 4 Improvement, methodology, query or suggestion
   2 ALERT type 5 Informative message, check
```

Validation response form

Please find below a validation response form (VRF) that can be filled in and pasted into your CIF.

```
# start Validation Reply Form
_vrf_PLAT910_preex1
;
PROBLEM: Missing # of FCF Reflection(s) Below Theta(Min)           6 Note
RESPONSE: ...
;
# end Validation Reply Form
```

It is advisable to attempt to resolve as many as possible of the alerts in all categories. Often the minor alerts point to easily fixed oversights, errors and omissions in your CIF or refinement strategy, so attention to these fine details can be worthwhile. In order to resolve some of the more serious problems it may be necessary to carry out additional measurements or structure refinements. However, the purpose of your study may justify the reported deviations and the more serious of these should normally be commented upon in the discussion or experimental section of a paper or in the "special_details" fields of the CIF. checkCIF was carefully designed to identify outliers and unusual parameters, but every test has its limitations and alerts that are not important in a particular case may appear. Conversely, the absence of alerts does not guarantee there are no aspects of the results needing attention. It is up to the individual to critically assess their own results and, if necessary, seek expert advice.

Publication of your CIF in IUCr journals

A basic structural check has been run on your CIF. These basic checks will be run on all CIFs submitted for publication in IUCr journals (*Acta Crystallographica*, *Journal of Applied Crystallography*, *Journal of Synchrotron Radiation*); however, if you intend to submit to *Acta Crystallographica Section C* or *E* or *IUCrData*, you should make sure that full publication checks are run on the final version of your CIF prior to submission.

Publication of your CIF in other journals

Please refer to the *Notes for Authors* of the relevant journal for any special instructions relating to CIF submission.

PLATON version of 27/03/2017; check.def file version of 24/03/2017

Datablock preex1 - ellipsoid plot

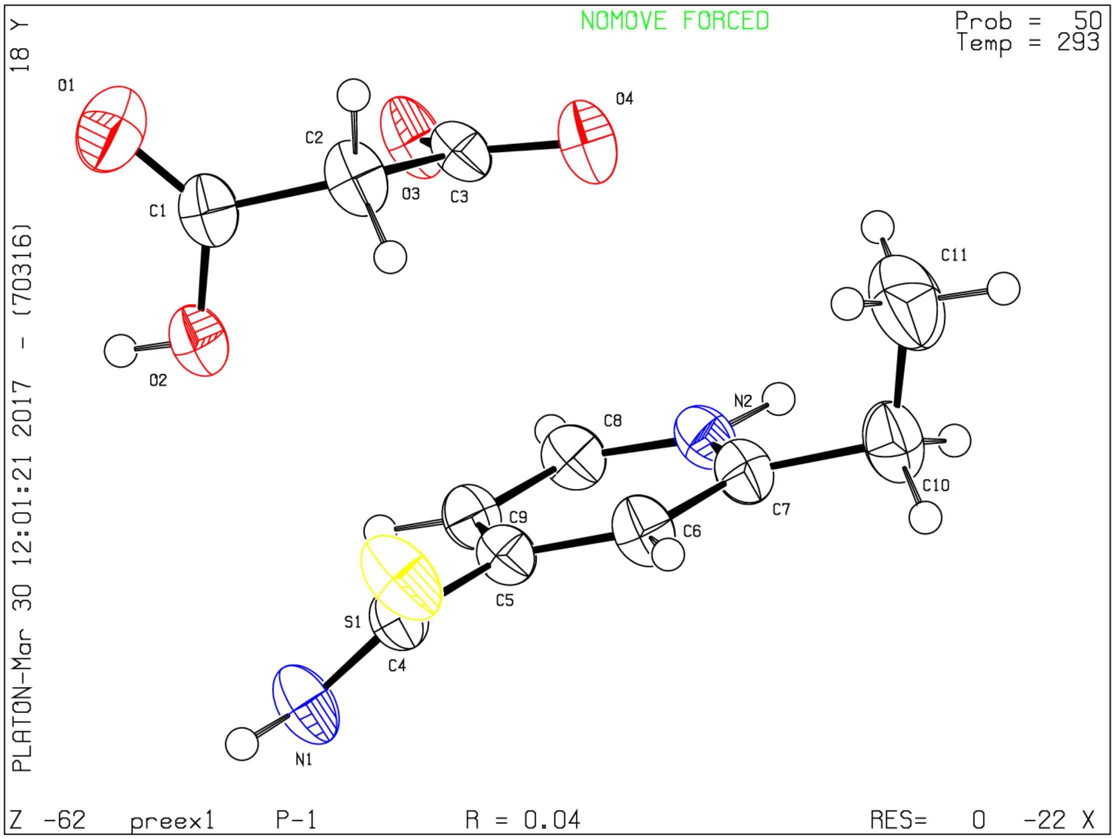

Supplement: Supplementary file 1 [file pharmaceutics-12-00818-s001.zip › pharmaceutics-900610-Supplementary/checkcif ETN-MAL.pdf]
