# Supplementary material for: Molecular Crystal Forms of Antitubercular Ethionamide with Dicarboxylic Acids: Solid-State Properties and a Combined Structural and Spectroscopic Study
Source: Pharmaceutics. 2020 Aug 28;12(9):818. doi: 10.3390/pharmaceutics12090818 (PMC7559828; doi:10.3390/pharmaceutics12090818)
Supplement: Supplementary file 1 [file pharmaceutics-12-00818-s001.zip › pharmaceutics-900610-Supplementary/pharmaceutics-900610 -Supplementary.pdf]

Supplementary Materials: Molecular Crystal Forms of Antitubercular Ethionamide with Dicarboxylic Acids: Solid-State Properties and a Combined Structural and Spectroscopic Study

Simone Bordignon, Paolo Cerreia Vioglio, Elena Amadio, Federica Rossi, Emanuele Priola, Dario Voinovich, Roberto Gobetto and Michele R. Chierotti

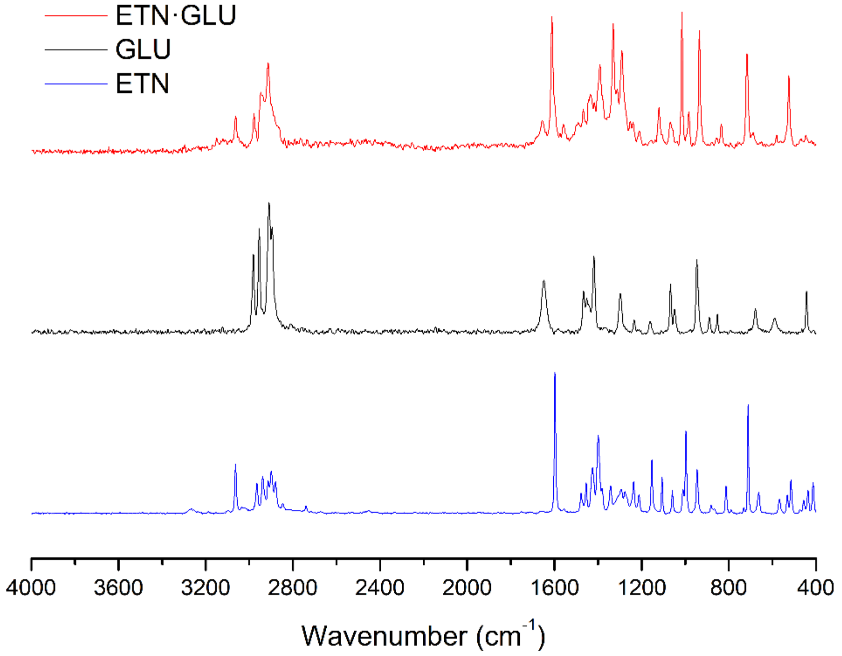

Figure S1. Comparison among Raman spectra of ETN (blue), GLU (black) and ETN·GLU (red).

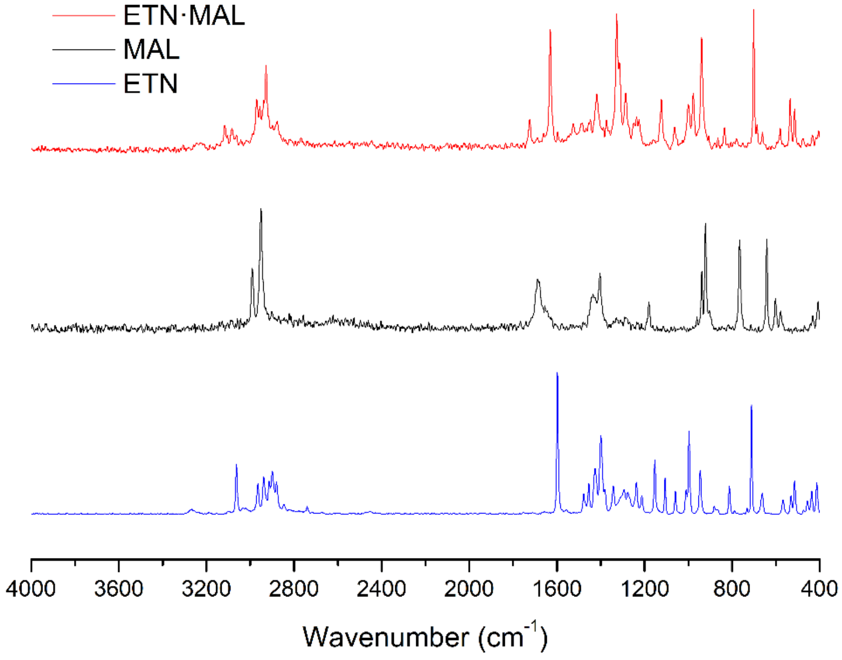

Figure S2. Comparison among Raman spectra of ETN (blue), MAL (black) and ETN·MAL (red).

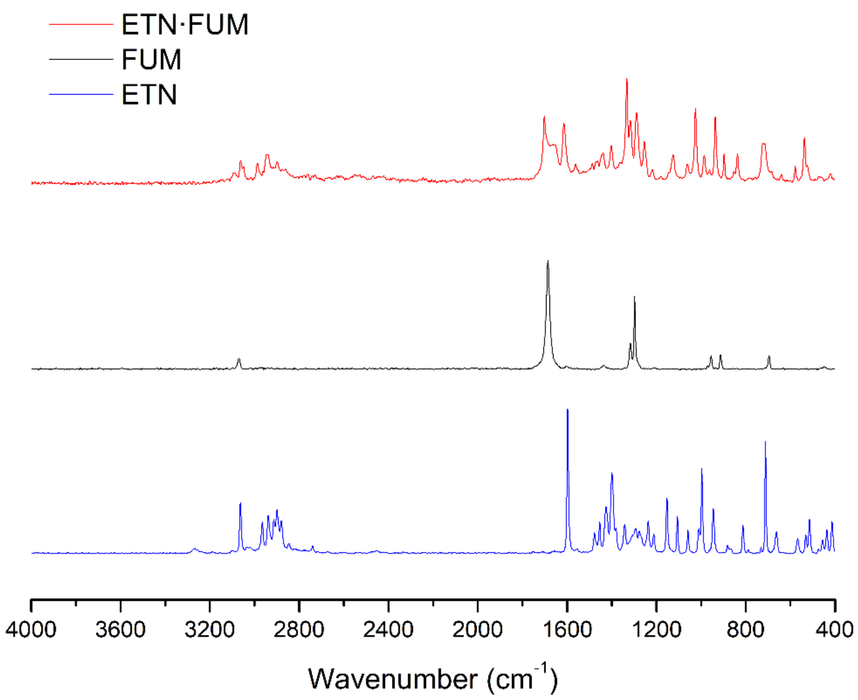

Figure S3. Comparison among Raman spectra of ETN (blue), FUM (black) and ETN·FUM (red).

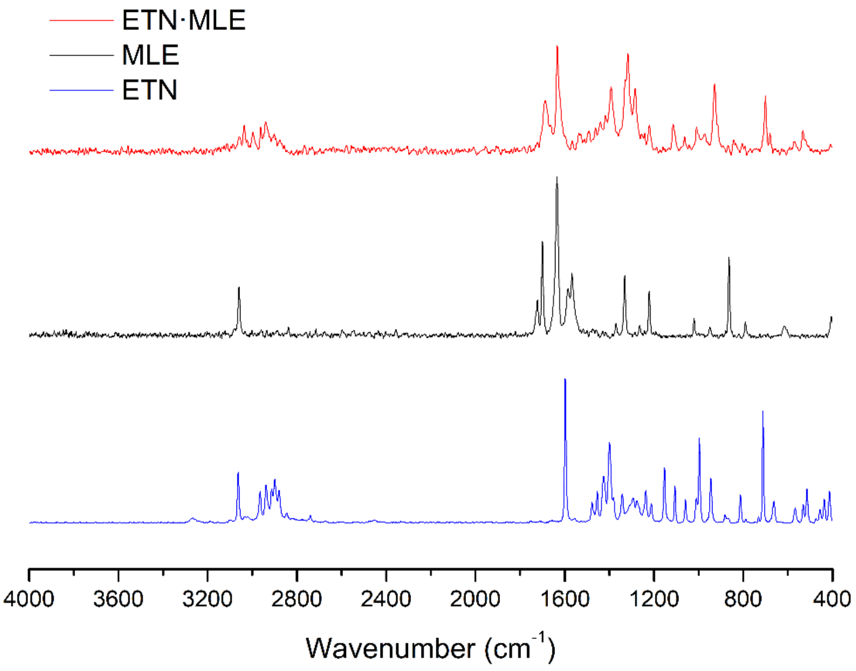

Figure S4. Comparison among Raman spectra of ETN (blue), MLE (black) and ETN·MLE (red).

Table S1. Values of distances between atoms in ETN·GLU X-ray structure. Refer to Scheme 1 in Main Text for atom numeration.

Atom	Atom	Length/Å	Atom	Atom	Length/Å
C4A	N5	1.345(12)	N5	C6B	1.358(13)
C4A	C3A	1.365(14)	C2	C7B	1.371(13)
C4A	C8A	1.561(11)	C2	C3A	1.377(13)
S11	C1	1.661(2)	C2	C3B	1.392(13)
O8'	C5'	1.217(2)	C2	C7A	1.407(12)
O9'	C5'	1.316(2)	C2	C1	1.494(3)
N10	C1	1.308(3)	C3'	C4'	1.504(3)
O6'	C1'	1.203(3)	C7A	C6A	1.381(14)
C2'	C1'	1.490(3)	C8A	C9A	1.479(17)
C2'	C3'	1.506(3)	C3B	C4B	1.391(14)
O7'	C1'	1.294(3)	C4B	C8B	1.624(17)
C5'	C4'	1.489(3)	C7B	C6B	1.368(14)
N5	C6A	1.330(12)	C8B	C9B	1.438(17)
N5	C4B	1.354(14)			

Table S2. Values of angles among atoms in ETN·GLU X-ray structure. Refer to Scheme 1 in Main Text for atom numeration.

Atom	Atom	Atom	Angle/°	Atom	Atom	Atom	Angle/°
N5	C4A	C3A	126.8(11)	C3B	C2	C1	123.4(7)
N5	C4A	C8A	112.0(9)	C7A	C2	C1	119.3(7)
C3A	C4A	C8A	120.5(10)	C4'	C3'	C2'	111.33(18)
C1'	C2'	C3'	117.35(18)	C5'	C4'	C3'	114.18(18)
O8'	C5'	O9'	122.6(2)	N10	C1	C2	116.23(18)
O8'	C5'	C4'	124.03(19)	N10	C1	S11	122.23(16)
O9'	C5'	C4'	113.34(19)	C2	C1	S11	121.54(15)
C6A	N5	C4A	112.9(7)	O6'	C1'	O7'	122.5(2)
C6A	N5	C4B	116.3(9)	O6'	C1'	C2'	122.02(19)
C4A	N5	C4B	16.4(13)	O7'	C1'	C2'	115.47(19)
C6A	N5	C6B	21.3(14)	C4A	C3A	C2	116.0(12)
C4A	N5	C6B	114.4(8)	N5	C6A	C7A	128.8(11)
C4B	N5	C6B	124.0(10)	C9A	C8A	C4A	109.9(10)
C7B	C2	C3A	114.9(10)	C4B	C3B	C2	126.4(13)
C7B	C2	C3B	112.8(10)	N5	C4B	C3B	114.4(13)
C3A	C2	C3B	16.0(10)	N5	C4B	C8B	121.5(13)
C7B	C2	C7A	19.3(13)	C3B	C4B	C8B	123.7(13)
C3A	C2	C7A	122.2(10)	C2	C7B	C6B	124.6(14)
C3B	C2	C7A	114.6(10)	N5	C6B	C7B	117.6(14)
C7B	C2	C1	123.7(7)	C9B	C8B	C4B	90.2(16)
C3A	C2	C1	118.5(7)				

Table S3. Values of distances between atoms in ETN·MAL X-ray structure. Refer to Scheme 1 in Main Text for atom numeration.

Atom	Atom	Length/Å	Atom	Atom	Length/Å
S11	C1	1.656(2)	C8	C4	1.500(3)
N5	C4	1.338(3)	C8	C9	1.506(4)
N5	C6	1.338(3)	O5'	C3'	1.234(3)
N10	C1	1.314(3)	O4'	C3'	1.264(3)
C6	C7	1.373(3)	O7'	C1'	1.327(3)
C3	C4	1.381(3)	O6'	C1'	1.198(3)
C3	C2	1.390(3)	C2'	C1'	1.497(3)
C7	C2	1.381(3)	C2'	C3'	1.530(3)
C2	C1	1.495(3)			

Table S4. Values of angles among atoms in ETN·MAL X-ray structure. Refer to Scheme 1 in Main Text for atom numeration.

Atom	Atom	Atom	Angle/°	Atom	Atom	Atom	Angle/°
C4	N5	C6	122.91(18)	N5	C4	C3	118.3(2)
N5	C6	C7	120.3(2)	N5	C4	C8	118.75(19)
C4	C3	C2	120.5(2)	C3	C4	C8	123.0(2)
C6	C7	C2	119.2(2)	C1'	C2'	C3'	114.00(18)
C7	C2	C3	118.85(19)	O5'	C3'	O4'	124.50(19)
C7	C2	C1	121.29(19)	O5'	C3'	C2'	117.23(19)
C3	C2	C1	119.9(2)	O4'	C3'	C2'	118.26(18)
C4	C8	C9	112.5(2)	O6'	C1'	O7'	124.1(2)
N10	C1	C2	116.21(19)	O6'	C1'	C2'	124.3(2)
N10	C1	S11	124.06(16)	O7'	C1'	C2'	111.6(2)
C2	C1	S11	119.73(15)				

Table S5. Values of distances between atoms in ETN·TAR X-ray structure. Refer to Scheme 1 in Main Text for atom numeration.

Atom	Atom	Length/Å	Atom	Atom	Length/Å
S11	C1	1.667(4)	C3A	C4A	1.386(6)
S11A	C1A	1.666(4)	C3	C4	1.391(6)
O5A’	C1A'	1.259(4)	C2A'	C3A'	1.533(6)
O10A’	C3A'	1.422(5)	C2A'	C1A'	1.532(6)
O7A’	C4A'	1.187(5)	C4	C8	1.503(6)
N5	C4	1.329(5)	C4A'	C3A'	1.521(6)
N5	C6	1.335(5)	C6A	C7A	1.367(6)
N5A	C6A	1.327(5)	C6	C7	1.369(6)
N5A	C4A	1.342(5)	C4A	C8A	1.517(6)
N10	C1	1.304(4)	C8A	C9A	1.438(10)
O8A’	C4A'	1.327(5)	C8	C9	1.465(10)
C2	C1	1.503(6)	O7'	C4'	1.209(5)
C2	C3	1.377(5)	O6'	C1'	1.292(4)
C2	C7	1.391(5)	O3'	C2'	1.405(5)
O9A’	C2A'	1.409(4)	O8'	C4'	1.327(5)
C2A	C1A	1.503(6)	O5'	C1'	1.213(4)
C2A	C3A	1.378(5)	C1'	C2'	1.523(6)
C2A	C7A	1.381(5)	C4'	C3'	1.511(6)
O6A’	C1A'	1.227(4)	O10'	C3'	1.396(5)
C1A	N10A	1.301(4)	C2'	C3'	1.541(6)

Table S6. Values of angles among atoms in ETN·TAR X-ray structure. Refer to Scheme 1 in Main Text for atom numeration.

Atom	Atom	Atom	Angle/°	Atom	Atom	Atom	Angle/°
C4	N5	C6	120.1(4)	O10A’	C3A'	C2A'	111.4(3)
C6A	N5A	C4A	121.5(4)	O10A’	C3A'	C4A'	108.1(3)
C3	C2	C1	120.0(3)	C4A'	C3A'	C2A'	109.3(3)
C3	C2	C7	117.8(4)	N5	C6	C7	122.5(4)
C7	C2	C1	122.2(3)	C6A	C7A	C2A	119.2(4)
C3A	C2A	C1A	120.5(3)	C6	C7	C2	118.9(4)
C3A	C2A	C7A	117.7(4)	O5A’	C1A'	C2A'	114.8(3)
C7A	C2A	C1A	121.8(3)	O6A’	C1A'	O5A’	127.3(4)
C2A	C1A	S11A	120.4(2)	O6A’	C1A'	C2A'	117.9(4)
N10A	C1A	S11A	122.8(4)	N5A	C4A	C3A	118.0(4)
N10A	C1A	C2A	116.8(3)	N5A	C4A	C8A	118.2(4)
N10	C1	S11	122.7(3)	C3A	C4A	C8A	123.8(4)
N10	C1	C2	116.8(3)	C9A	C8A	C4A	112.4(6)
C2	C1	S11	120.5(3)	C9	C8	C4	111.4(6)
C2A	C3A	C4A	121.7(4)	O6'	C1'	C2'	112.4(3)
C2	C3	C4	120.7(4)	O5'	C1'	O6'	124.5(4)
O9A’	C2A'	C3A'	110.1(3)	O5'	C1'	C2'	123.1(3)
O9A’	C2A'	C1A'	112.7(3)	O7'	C4'	O8'	124.7(4)
C1A'	C2A'	C3A'	110.2(3)	O7'	C4'	C3'	123.8(4)
N5	C4	C3	120.1(3)	O8'	C4'	C3'	111.4(4)
N5	C4	C8	117.5(4)	O3'	C2'	C1'	109.4(3)
C3	C4	C8	122.4(4)	O3'	C2'	C3'	111.4(3)
O7A’	C4A'	O8A’	124.4(4)	C1'	C2'	C3'	107.9(3)
O7A’	C4A'	C3A'	126.3(4)	C4'	C3'	C2'	109.1(3)
O8A’	C4A'	C3A'	109.3(4)	O10'	C3'	C4'	111.5(3)
N5A	C6A	C7A	121.8(4)	O10'	C3'	C2'	112.1(4)

Table S7. NMR parameters employed for all SSNMR experiments.

	^{13}C CPMAS spectra								
	ETN	GLU	MAL	FUM	MLE	ETN·GLU	ETN·MAL	ETN·FUM	ETN·MLE
n° scans	216	24	48	24	4	170	1184	609	50
relaxation delay (s)	2.8	73	50	211.2	360	4.8	1.8	2.5	9.7
contact time (ms)	3	3	3	3	5	3	3	3	3
acquisition time (ms)	32	32	55	32	30	32	35	35	32
n° points	2348	2348	3672	2348	2722	2348	2568	2568	2348
spectral width (kHz)	36.8	37	36.8	36.8	45	36.8	36.8	36.8	36.8
	^{15}N CPMAS spectra								
n° scans	19932	/	/	/	/	1937	531	21932	5892
relaxation delay (s)	2.8	/	/	/	/	4.8	1.8	2.5	9.7
contact time (ms)	4	/	/	/	/	4	4	3	4
acquisition time (ms)	39	/	/	/	/	39	39	39	39
n° points	2048	/	/	/	/	2048	2048	2048	2048
spectral width (kHz)	26.3	/	/	/	/	26.3	26.3	26.3	26.3

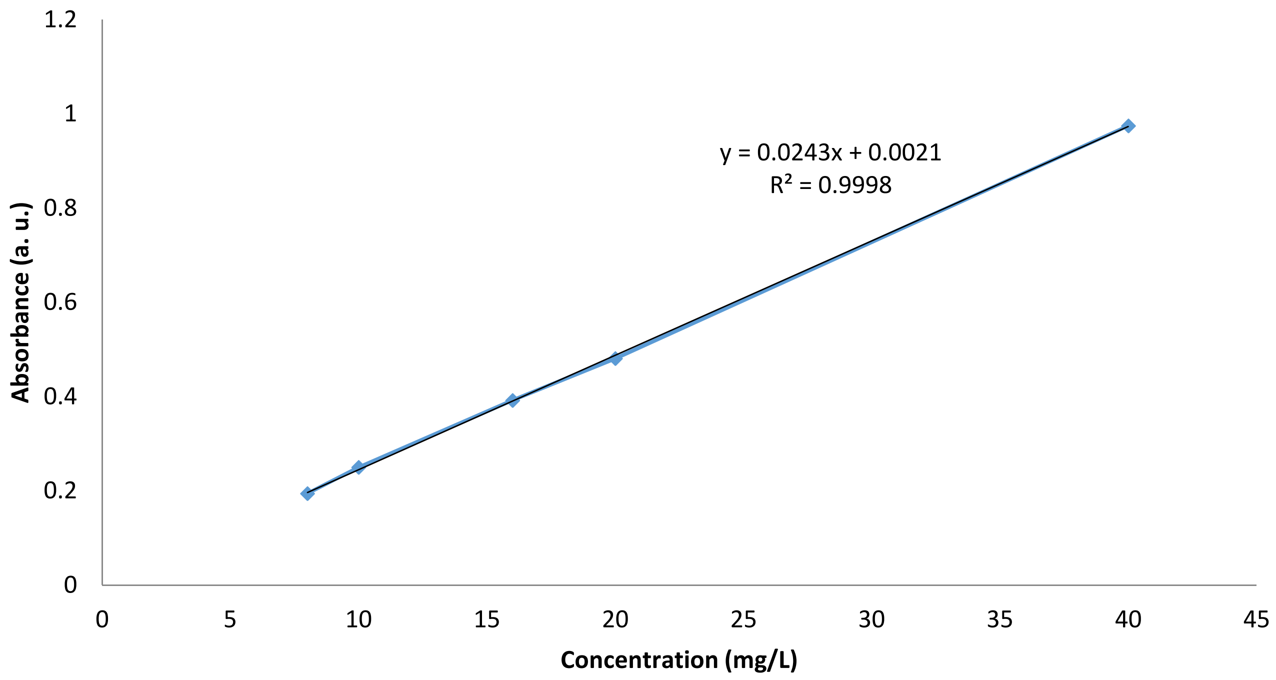

Figure S5. Calibration curve for dissolution kinetic tests with correspondent equation for ETN.

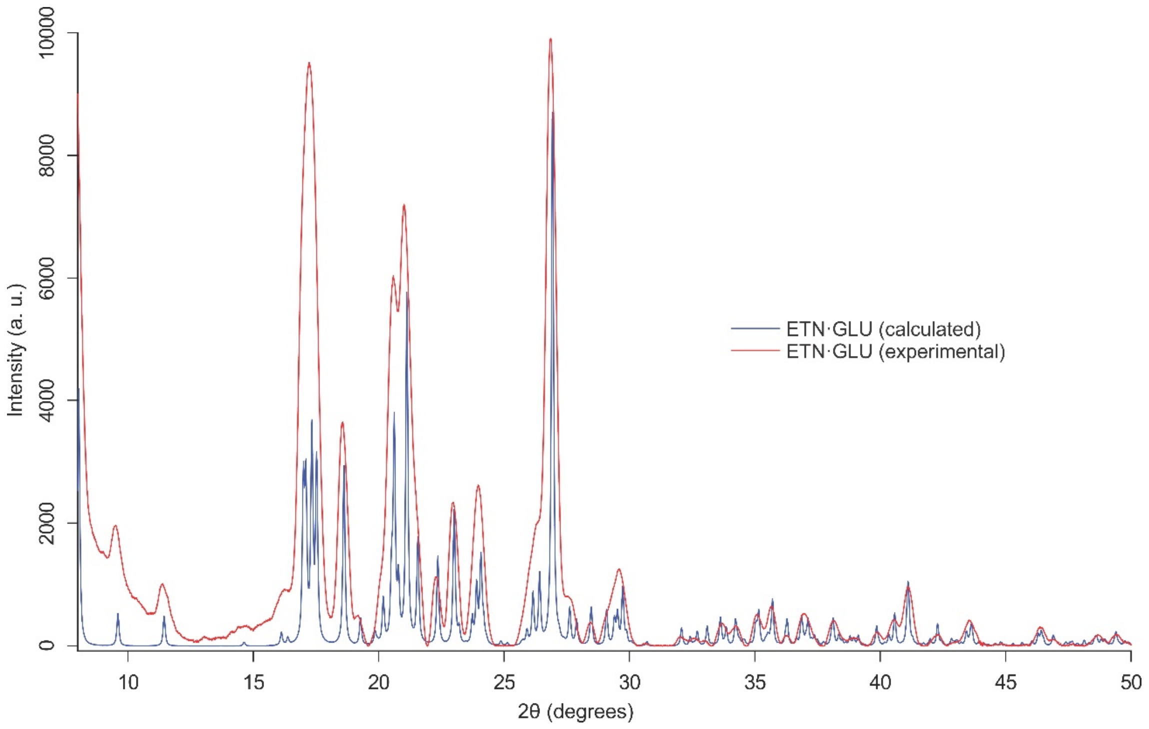

Figure S6. Comparison between calculated (blue) and experimental (red) PXRD patterns for ETN·GLU.

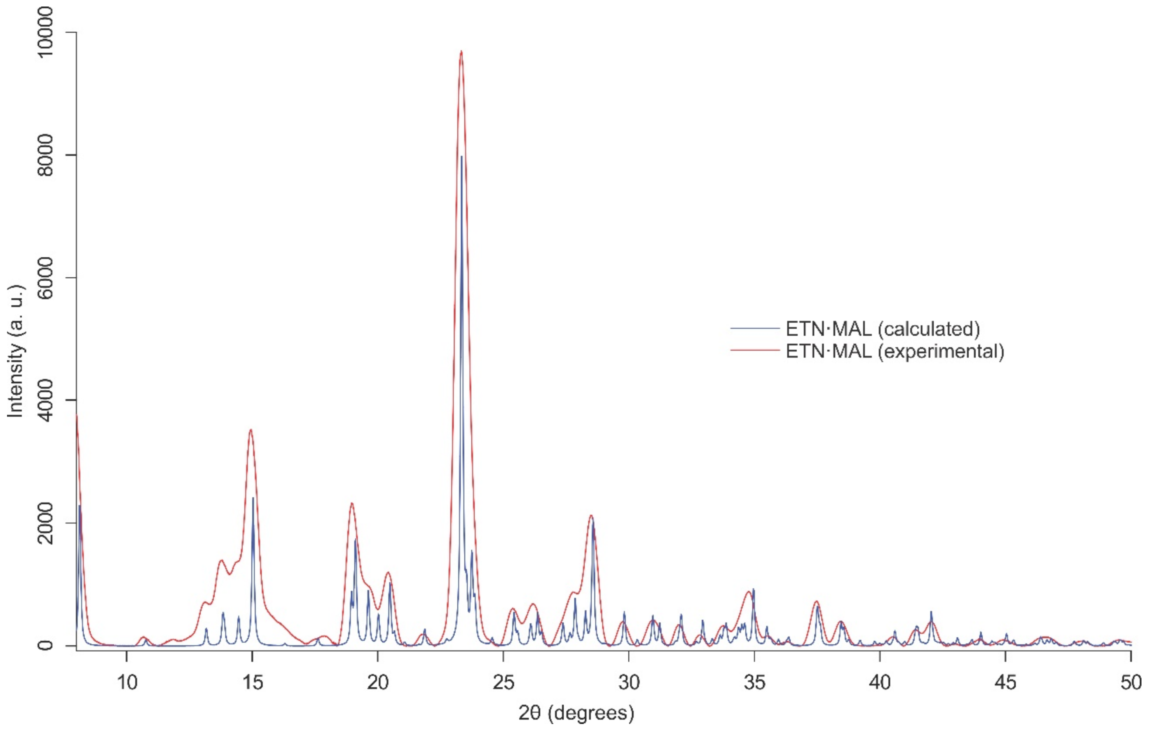

Figure S7. Comparison between calculated (blue) and experimental (red) PXRD patterns for ETN·MAL.

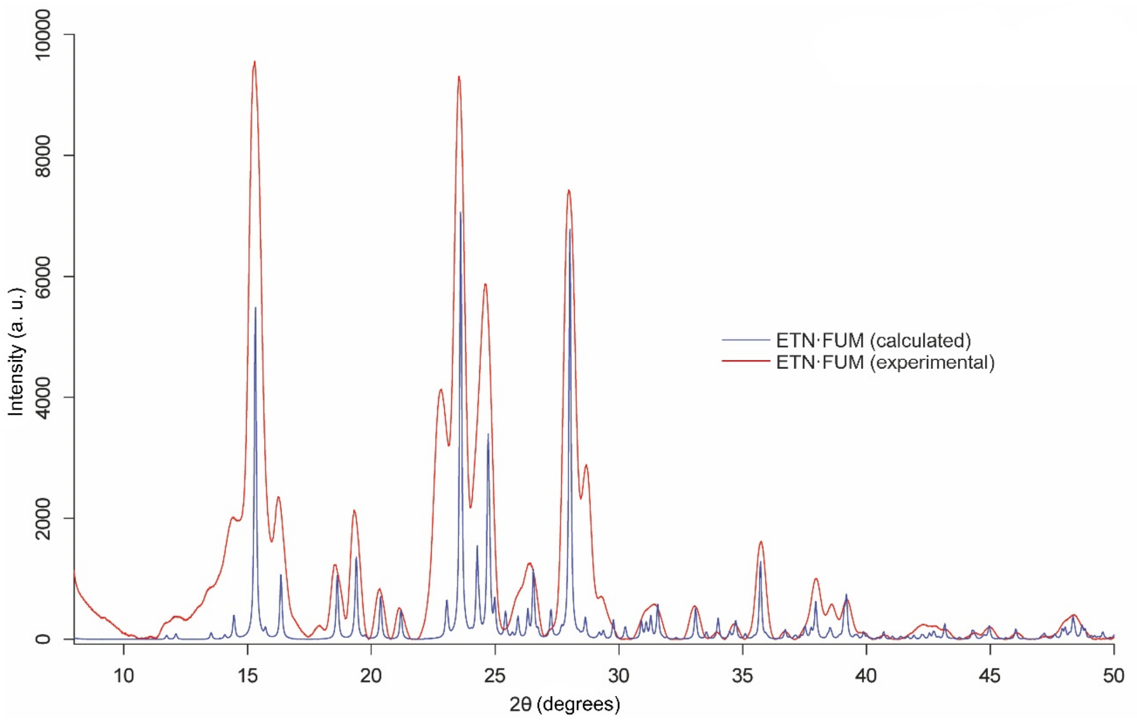

Figure S8. Comparison between calculated (blue) and experimental (red) PXRD patterns for ETN·FUM.

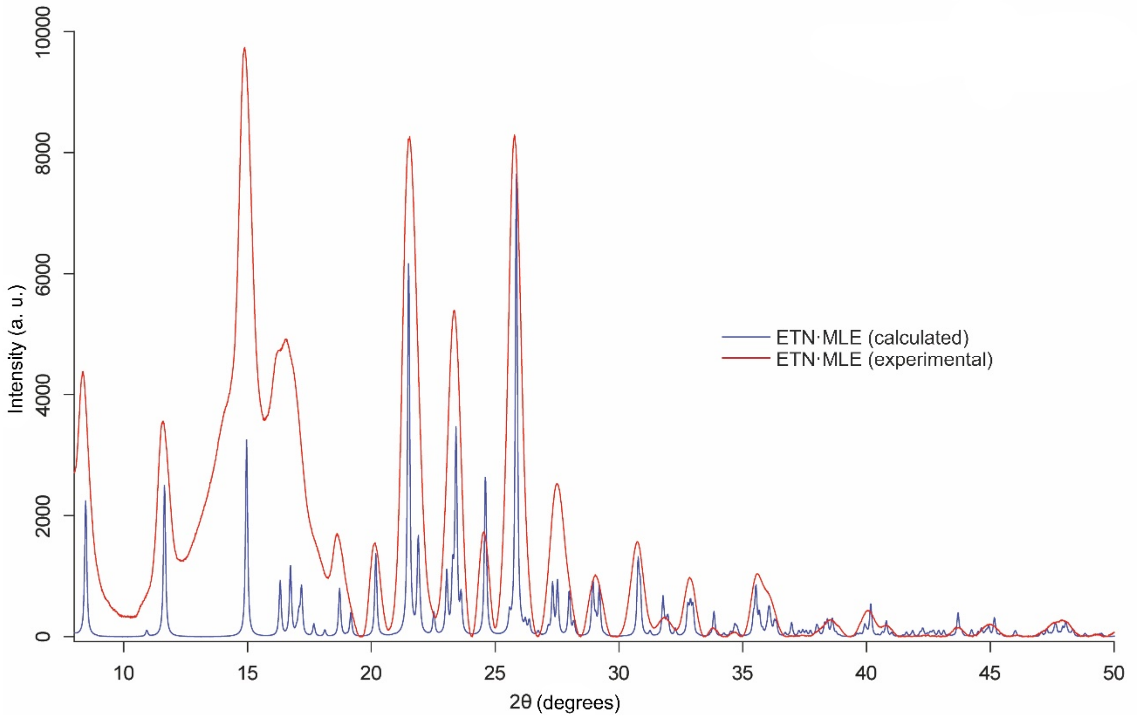

Figure S9. Comparison between calculated (blue) and experimental (red) PXRD patterns for ETN·MLE.

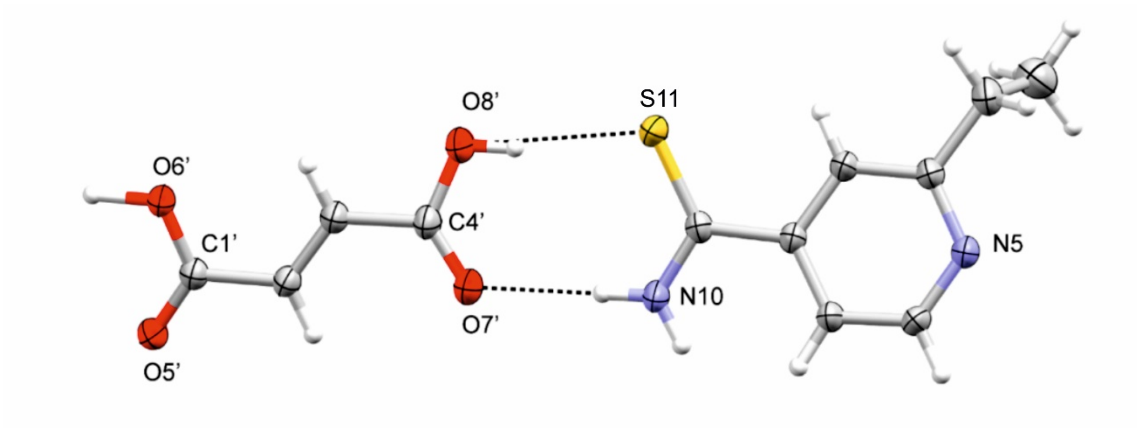

Figure S10. Asymmetric unit of ETN·FUM.

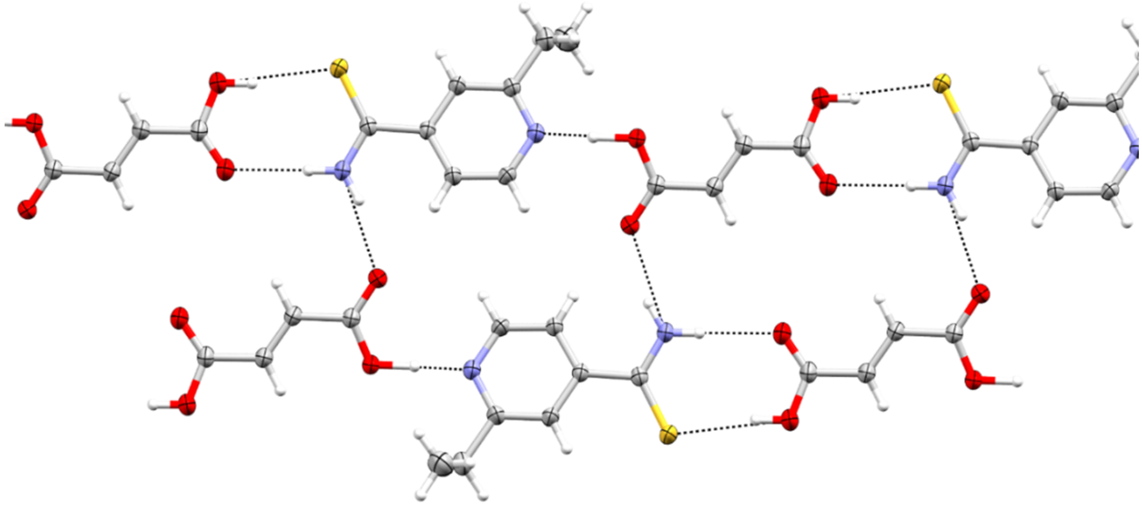

Figure S11. HB pattern of ETN·FUM.

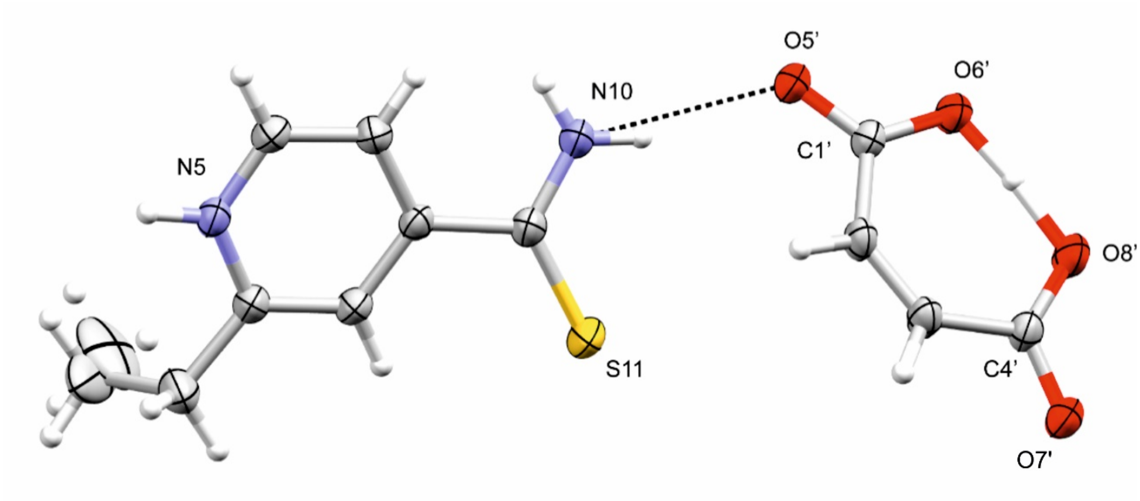

Figure S12. Asymmetric unit of ETN·MLE.

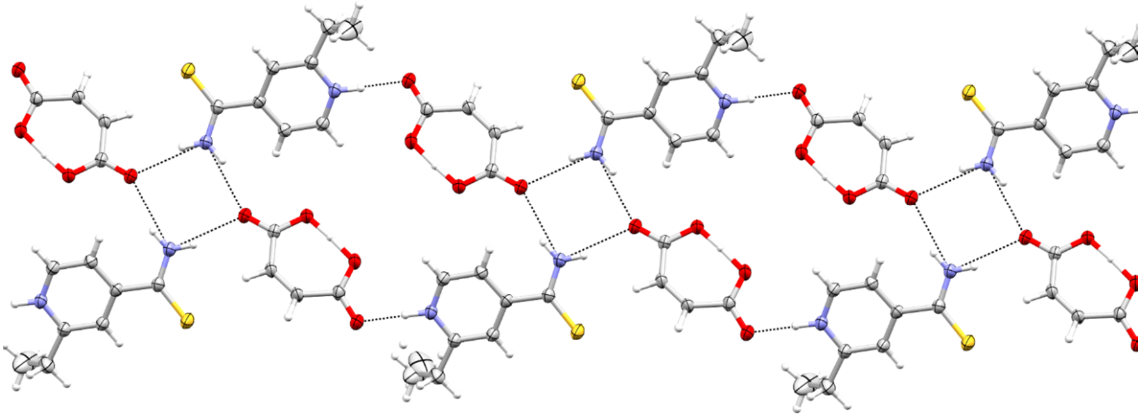

Figure S13. HB pattern of ETN·MLE.

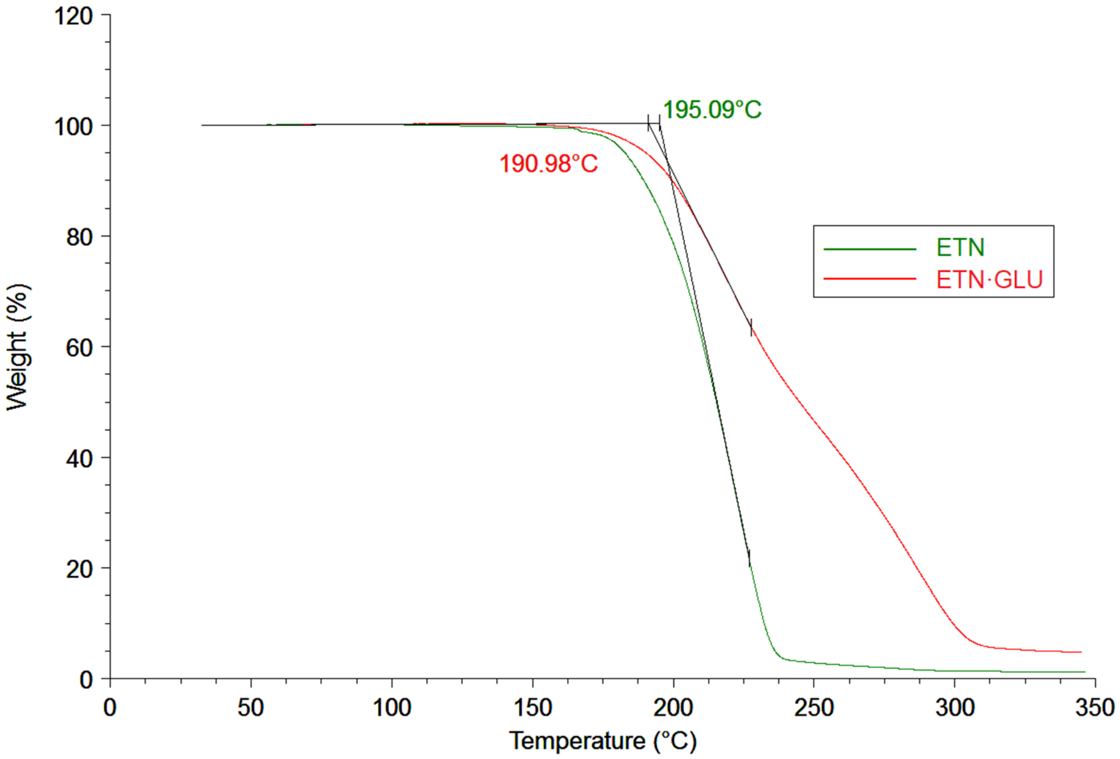

Figure S14. Comparison between TGA curves of ETN (green) and ETN·GLU (red).

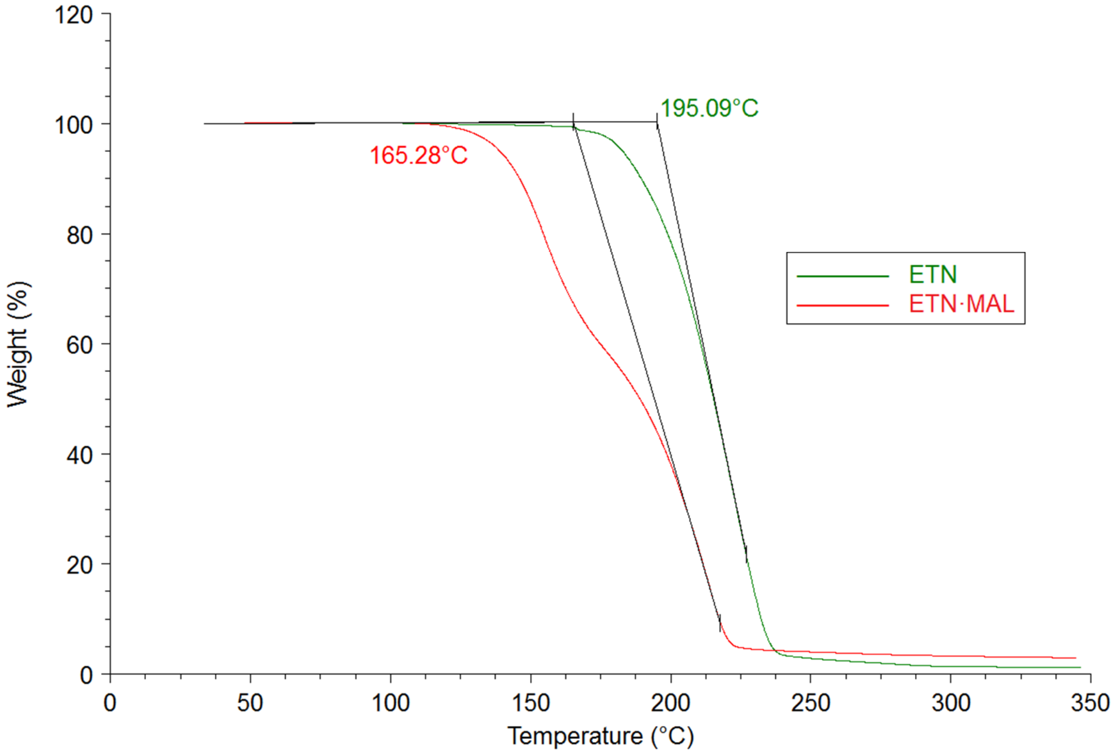

Figure S15. Comparison between TGA curves of ETN (green) and ETN·MAL (red).

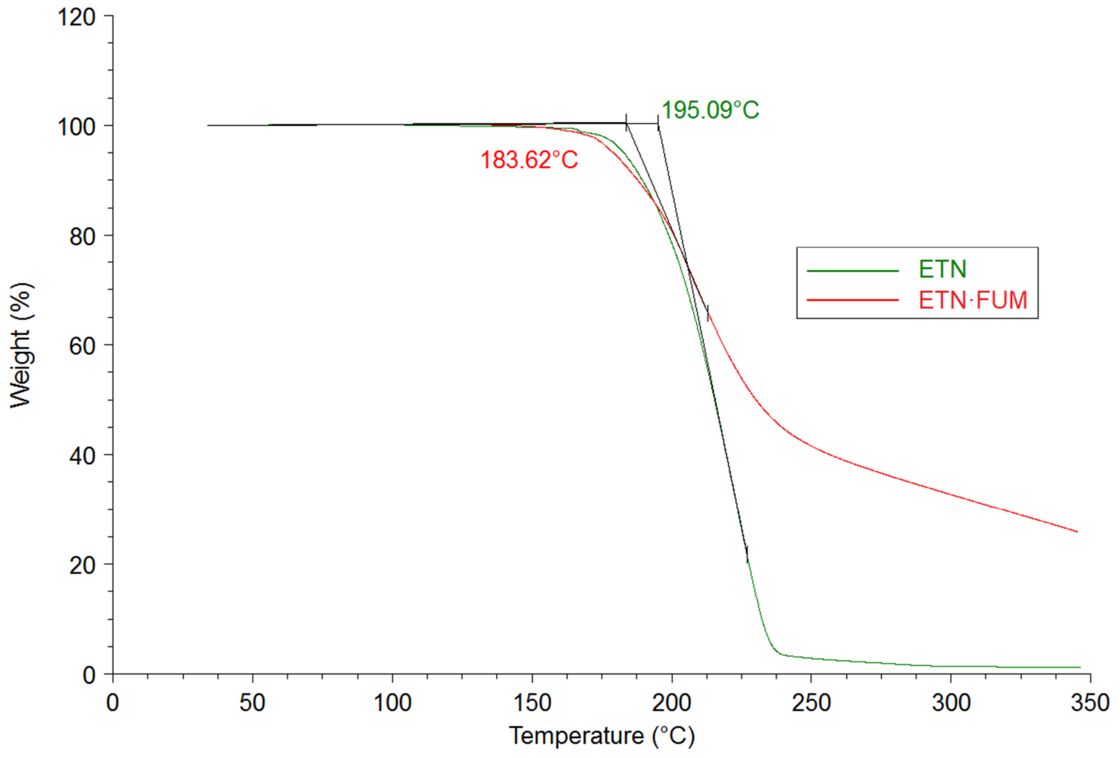

Figure S16. Comparison between TGA curves of ETN (green) and ETN·FUM (red).

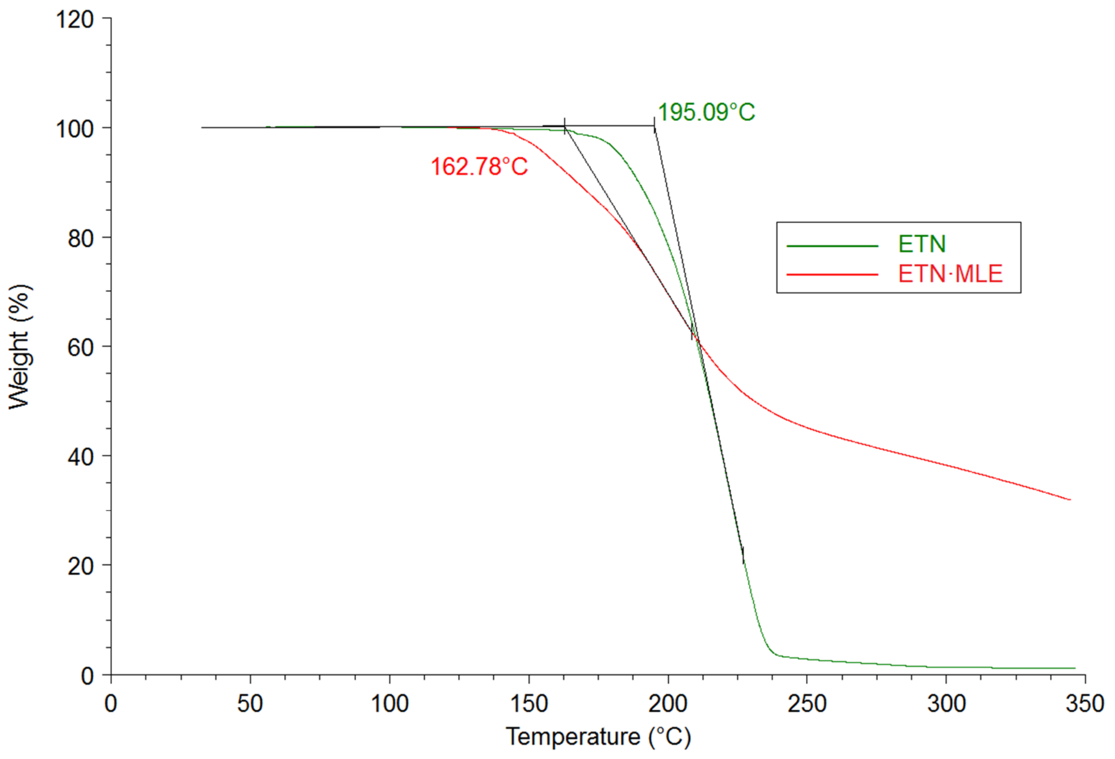

Figure S17. Comparison between TGA curves of ETN (green) and ETN·MLE (red).

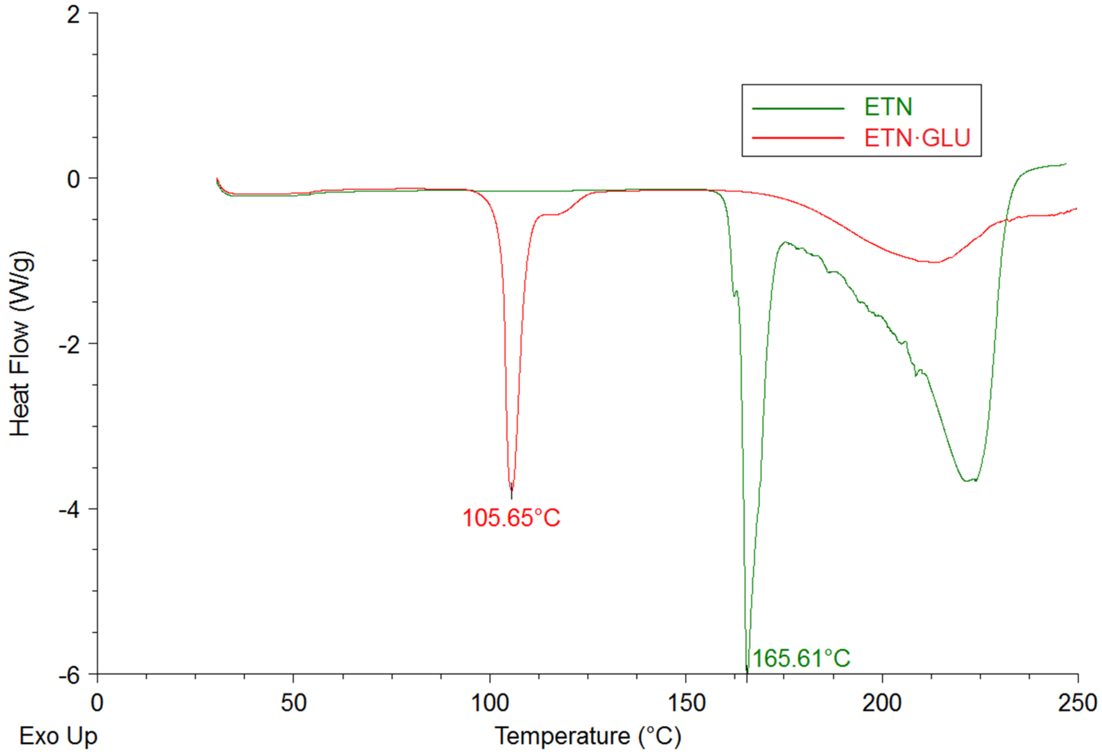

Figure S18. Comparison between DSC curves of ETN (green) and ETN·GLU (red).

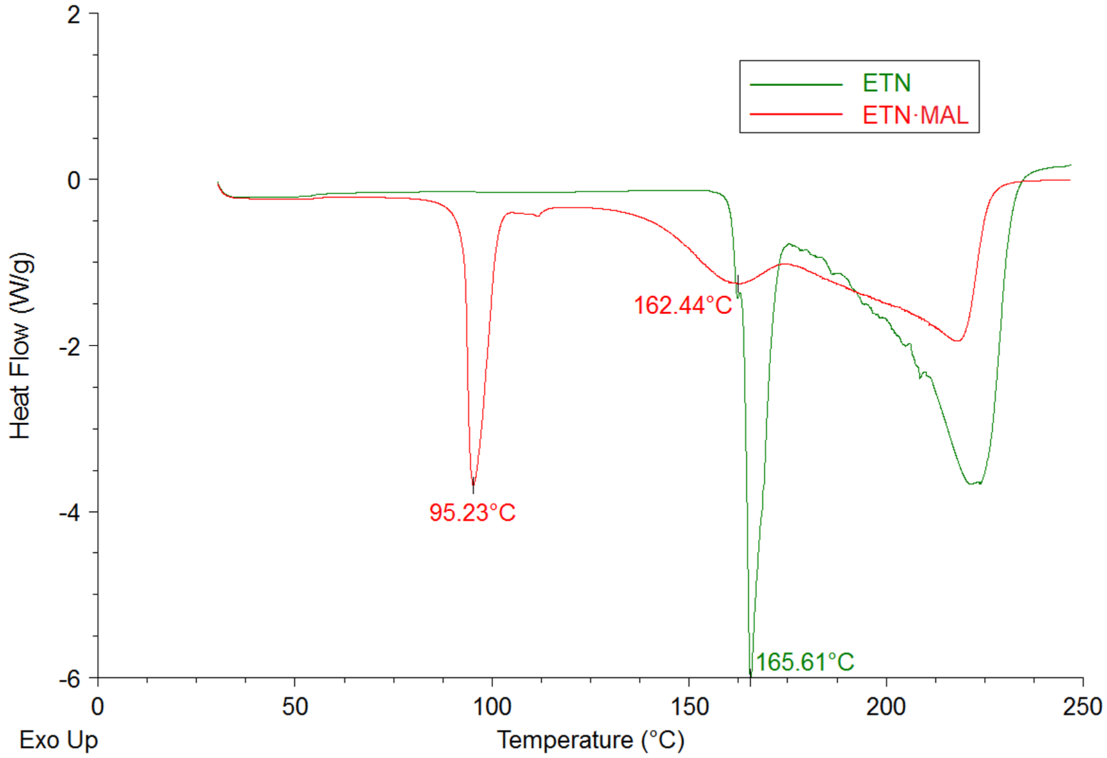

Figure S19. Comparison between DSC curves of ETN (green) and ETN·MAL (red).

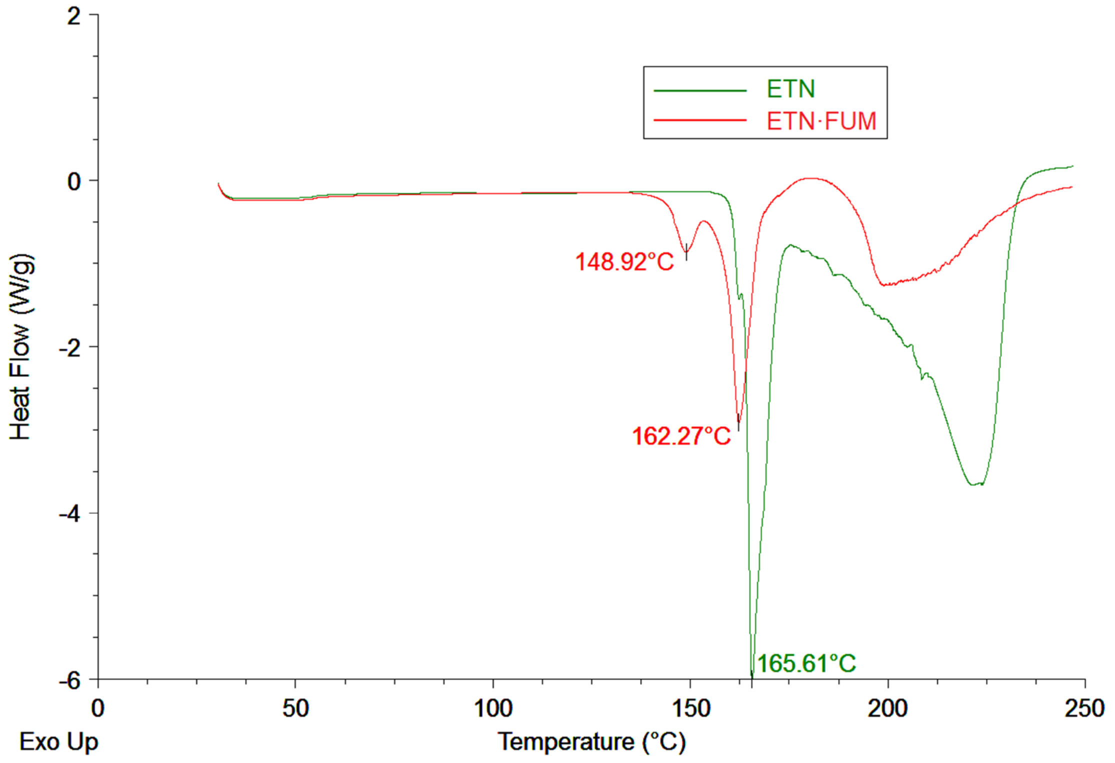

Figure S20. Comparison between DSC curves of ETN (green) and ETN·FUM (red).

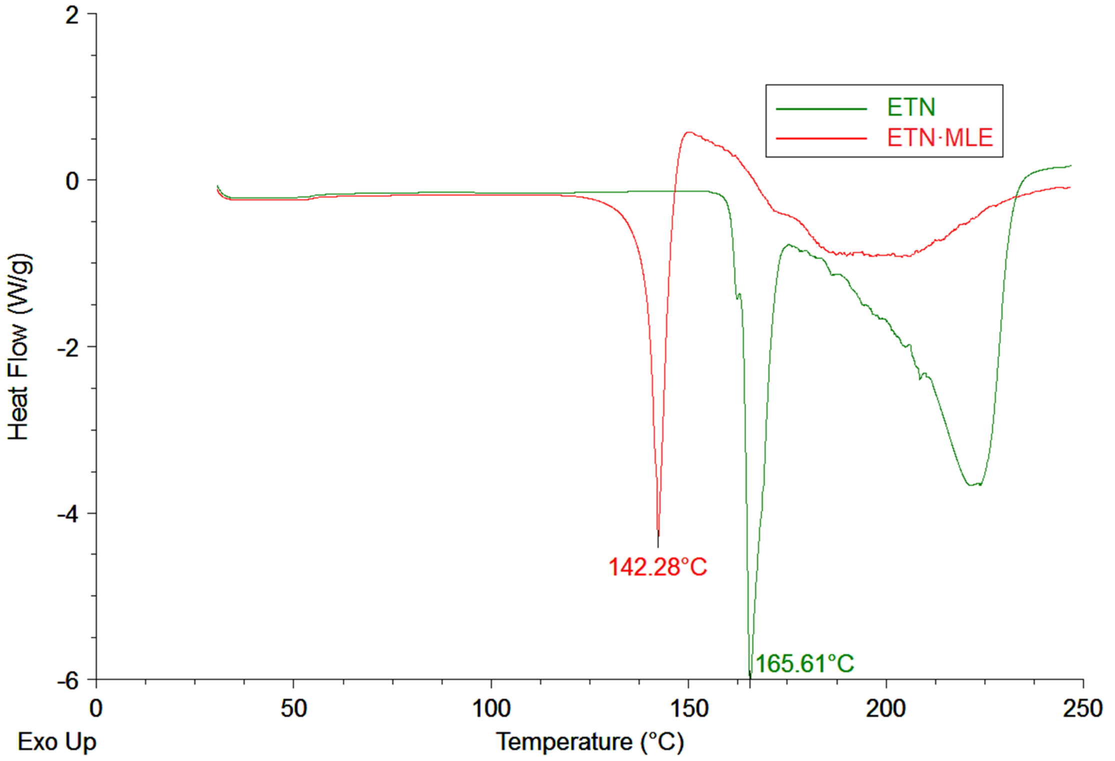

Figure S21. Comparison between DSC curves of ETN (green) and ETN·MLE (red).
