# Supplementary material for: Molecular Crystal Forms of Antitubercular Ethionamide with Dicarboxylic Acids: Solid-State Properties and a Combined Structural and Spectroscopic Study
Source: Pharmaceutics. 2020 Aug 28;12(9):818. doi: 10.3390/pharmaceutics12090818 (PMC7559828; doi:10.3390/pharmaceutics12090818)

checkCIF/PLATON report

You have not supplied any structure factors. As a result the full set of tests cannot be run.

THIS REPORT IS FOR GUIDANCE ONLY. IF USED AS PART OF A REVIEW PROCEDURE FOR PUBLICATION, IT SHOULD NOT REPLACE THE EXPERTISE OF AN EXPERIENCED CRYSTALLOGRAPHIC REFEREE.

No syntax errors found. CIF dictionary Interpreting this report

Datablock: preex6

```
Bond precision:  C-C = 0.0030 A          Wavelength=0.71073

Cell:          a=5.3818(3)       b=11.4336(6)     c=13.2901(9)
               alpha=78.196(5)   beta=80.618(5)   gamma=77.435(5)
Temperature:   293 K

                Calculated                Reported
Volume          775.29(8)                 775.28(8)
Space group     P -1                      P-1
Hall group      -P 1                      ?
Moiety formula  C8 H10 N2 S, C5 H8 O4     ?
Sum formula     C13 H18 N2 O4 S           C13 H18 N2 O4 S
Mr              298.35                    298.35
Dx,g cm-3       1.278                     1.278
Z               2                         2
Mu (mm-1)       0.222                     0.222
F000            316.0                     316.0
F000'           316.40
h,k,lmax        6,13,15                   6,13,15
Nref            2744                      2739
Tmin,Tmax       0.943,0.954               0.941,1.000
Tmin'           0.895

Correction method= # Reported T Limits: Tmin=0.941 Tmax=1.000
AbsCorr = MULTI-SCAN

Data completeness= 0.998          Theta(max)= 25.030

R(reflections)= 0.0448( 2132)     wR2(reflections)= 0.1236( 2739)

S = 1.043                    Npar= 238
```

The following ALERTS were generated. Each ALERT has the format
test-name_ALERT_alert-type_alert-level.
Click on the hyperlinks for more details of the test.

Alert level B

```
PLAT213_ALERT_2_B Atom C7A            has ADP max/min Ratio .....        4.2 prolat
```

Author Response: strong disorder of rotation arround C1-C2

Alert level C

```
PLAT213_ALERT_2_C Atom C8B            has ADP max/min Ratio .....        3.1 prolat
```

Author Response: strong disorder of rotation arround C1-C2

```
PLAT220_ALERT_2_C Non-Solvent Resd 1   C   Ueq(max)/Ueq(min) Range        4.2 Ratio
```

Author Response: strong disorder of rotation arround C1-C2

```
PLAT222_ALERT_3_C Non-Solvent Resd 1   H Uiso(max)/Uiso(min) Range        4.8 Ratio
```

Author Response: strong disorder of rotation arround C1-C2

```
PLAT242_ALERT_2_C Low    'MainMol' Ueq as Compared to Neighbors of         C9 Check
PLAT250_ALERT_2_C Large U3/U1 Ratio for Average U(i,j) Tensor ....        3.1 Note
```

Author Response: strong disorder of rotation arround C1-C2

```
PLAT250_ALERT_2_C Large U3/U1 Ratio for Average U(i,j) Tensor ....        3.6 Note
```

Author Response: strong disorder of rotation arround C1-C2

```
PLAT414_ALERT_2_C Short Intra D-H..H-X       H1A    ..  H6B     ..       1.93 Ang.
```

Alert level G

```
PLAT002_ALERT_2_G Number of Distance or Angle Restraints on AtSite         14 Note
PLAT005_ALERT_5_G No Embedded Refinement Details found  in the CIF     Please Do !
PLAT007_ALERT_5_G Number of Unrefined Donor-H Atoms .............          4 Report
PLAT093_ALERT_1_G No s.u.'s on H-positions, Refinement Reported as      mixed Check
PLAT154_ALERT_1_G The s.u.'s on the Cell Angles are Equal ..(Note)      0.005 Degree
PLAT199_ALERT_1_G Reported _cell_measurement_temperature ..... (K)        293 Check
PLAT200_ALERT_1_G Reported   _diffrn_ambient_temperature ..... (K)        293 Check
PLAT301_ALERT_3_G Main Residue  Disorder .............(Resd  1)..         55 % Note
PLAT720_ALERT_4_G Number of Unusual/Non-Standard Labels ..........         10 Note
PLAT779_ALERT_4_G Suspect or Irrelevant (Bond) Angle in CIF .... #         10 Check
              C4A  -N2   -C4B     1.555   1.555   1.555            16.40 Deg.
PLAT779_ALERT_4_G Suspect or Irrelevant (Bond) Angle in CIF .... #         11 Check
              C5A  -N2   -C5B     1.555   1.555   1.555            21.30 Deg.
PLAT779_ALERT_4_G Suspect or Irrelevant (Bond) Angle in CIF .... #         16 Check
              C3A  -C2   -C3B     1.555   1.555   1.555            16.00 Deg.
```

```
PLAT779_ALERT_4_G Suspect or Irrelevant (Bond) Angle in CIF .... #         17 Check
              C6B  -C2   -C6A     1.555   1.555   1.555            19.30 Deg.
PLAT811_ALERT_5_G No ADDSYM Analysis: Too Many Excluded Atoms ....          ! Info
PLAT860_ALERT_3_G Number of Least-Squares Restraints .............         25 Note
PLAT899_ALERT_4_G SHELXL97   is Deprecated and Succeeded by SHELXL       2014 Note
```

```
   0 ALERT level A = Most likely a serious problem - resolve or explain
   1 ALERT level B = A potentially serious problem, consider carefully
   7 ALERT level C = Check. Ensure it is not caused by an omission or oversight
  16 ALERT level G = General information/check it is not something unexpected

   4 ALERT type 1 CIF construction/syntax error, inconsistent or missing data
   8 ALERT type 2 Indicator that the structure model may be wrong or deficient
   3 ALERT type 3 Indicator that the structure quality may be low
   6 ALERT type 4 Improvement, methodology, query or suggestion
   3 ALERT type 5 Informative message, check
```

Validation response form

Please find below a validation response form (VRF) that can be filled in and pasted into your CIF.

```
# start Validation Reply Form
_vrf_PLAT242_preex6
;
PROBLEM: Low    ’MainMol’ Ueq as Compared to Neighbors of         C9 Check
RESPONSE: ...
;
_vrf_PLAT414_preex6
;
PROBLEM: Short Intra D-H..H-X       H1A    ..  H6B     ..       1.93 Ang.
RESPONSE: ...
;
# end Validation Reply Form
```

It is advisable to attempt to resolve as many as possible of the alerts in all categories. Often the minor alerts point to easily fixed oversights, errors and omissions in your CIF or refinement strategy, so attention to these fine details can be worthwhile. In order to resolve some of the more serious problems it may be necessary to carry out additional measurements or structure refinements. However, the purpose of your study may justify the reported deviations and the more serious of these should normally be commented upon in the discussion or experimental section of a paper or in the "special_details" fields of the CIF. checkCIF was carefully designed to identify outliers and unusual parameters, but every test has its limitations and alerts that are not important in a particular case may appear. Conversely, the absence of alerts does not guarantee there are no aspects of the results needing attention. It is up to the individual to critically assess their own results and, if necessary, seek expert advice.

Publication of your CIF in IUCr journals

A basic structural check has been run on your CIF. These basic checks will be run on all CIFs submitted for publication in IUCr journals (*Acta Crystallographica*, *Journal of Applied Crystallography*, *Journal of Synchrotron Radiation*); however, if you intend to submit to *Acta Crystallographica Section C* or *E* or *IUCrData*, you should make sure that full publication checks are run on the final version of your CIF prior to submission.

Publication of your CIF in other journals

Please refer to the *Notes for Authors* of the relevant journal for any special instructions relating to CIF submission.

PLATON version of 26/02/2017; check.def file version of 21/02/2017

Datablock preex6 - ellipsoid plot

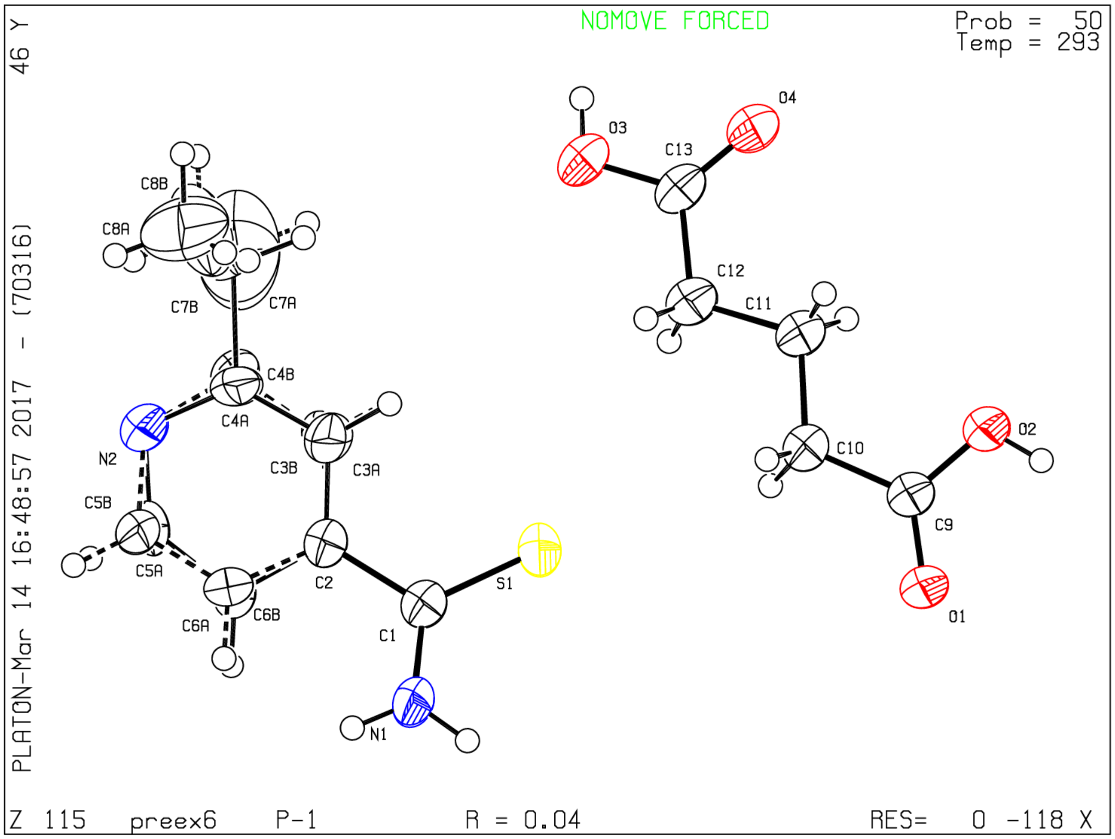

Supplement: Supplementary file 1 [file checkcif ETN-GLU.pdf › pharmaceutics-900610-Supplementary/checkcif ETN-GLU.pdf]
