# Supplementary material for: Molecular Crystal Forms of Antitubercular Ethionamide with Dicarboxylic Acids: Solid-State Properties and a Combined Structural and Spectroscopic Study
Source: Pharmaceutics. 2020 Aug 28;12(9):818. doi: 10.3390/pharmaceutics12090818 (PMC7559828; doi:10.3390/pharmaceutics12090818)

checkCIF/PLATON report

You have not supplied any structure factors. As a result the full set of tests cannot be run.

THIS REPORT IS FOR GUIDANCE ONLY. IF USED AS PART OF A REVIEW PROCEDURE FOR PUBLICATION, IT SHOULD NOT REPLACE THE EXPERTISE OF AN EXPERIENCED CRYSTALLOGRAPHIC REFEREE.

No syntax errors found.   CIF dictionary   Interpreting this report

Datablock: preex3a

```
Bond precision:  C-C = 0.0053 A          Wavelength=0.71073

Cell:            a=4.8561(3)     b=24.2003(13)      c=12.1304(6)
                 alpha=90        beta=92.671(5)     gamma=90
Temperature:     293 K
```

	Calculated	Reported
Volume	1424.01(14)	1424.00(14)
Space group	P 21	P2(1)
Hall group	P 2yb	?
Moiety formula	C12 H16 N2 O6 S, C8 H11 N2 S, C4 H5 O6	?
Sum formula	C24 H32 N4 O12 S2	C24 H32 N4 O12 S2
Mr	632.66	632.66
Dx,g cm-3	1.475	1.475
Z	2	2
Mu (mm-1)	0.257	0.257
F000	664.0	664.0
F000’	664.86	
h,k,lmax	5,28,14	5,28,14
Nref	5020[2574]	4925
Tmin,Tmax	0.898,0.947	0.986,1.000
Tmin’	0.898	

```
Correction method= # Reported T Limits: Tmin=0.986 Tmax=1.000
AbsCorr = MULTI-SCAN

Data completeness= 1.91/0.98      Theta(max)= 25.020

R(reflections)= 0.0475( 3977)     wR2(reflections)= 0.0918( 4925)

S = 1.036                  Npar= 395
```

The following ALERTS were generated. Each ALERT has the format
test-name_ALERT_alert-type_alert-level.
Click on the hyperlinks for more details of the test.

Alert level A

PLAT353_ALERT_3_A Long N-H (N0.87,N1.01A) N2 - H2A .. 1.35 Ang.

Author Response: disorder in the position of hydrogen bond due to resonance

---

Alert level B

PLAT353_ALERT_3_B Long N-H (N0.87,N1.01A) N4 - H4C .. 1.12 Ang.

Author Response: disorder in the position of hydrogen bond due to resonance

---

Alert level C

PLAT089_ALERT_3_C Poor Data / Parameter Ratio (Zmax < 18) .... 6.52 Note
PLAT220_ALERT_2_C Non-Solvent Resd 1 C Ueq(max)/Ueq(min) Range 3.5 Ratio

Author Response: disorder in methyl group

PLAT222_ALERT_3_C Non-Solvent Resd 1 H Uiso(max)/Uiso(min) Range 4.3 Ratio

Author Response: disorder in methyl group

PLAT250_ALERT_2_C Large U3/U1 Ratio for Average U(i,j) Tensor .... 2.3 Note

Author Response: disorder in methyl group

PLAT250_ALERT_2_C Large U3/U1 Ratio for Average U(i,j) Tensor .... 2.3 Note

Author Response: disorder in methyl group

PLAT250_ALERT_2_C Large U3/U1 Ratio for Average U(i,j) Tensor .... 2.3 Note

Author Response: disorder in methyl group

PLAT303_ALERT_2_C Full Occupancy H-Atom H2A with # Connections 2.00 Check
PLAT340_ALERT_3_C Low Bond Precision on C-C Bonds .... 0.00525 Ang.
PLAT414_ALERT_2_C Short Intra D-H..H-X H1A .. H3 .. 1.94 Ang.

Author Response: conformation of molecule

```
PLAT414_ALERT_2_C Short Intra D-H..H-X       H3A    ..  H15     ..       1.93 Ang.
```

Author Response: conformation of molecule

Alert level G

```
PLAT005_ALERT_5_G No Embedded Refinement Details found  in the CIF     Please Do !
PLAT007_ALERT_5_G Number of Unrefined Donor-H Atoms .............         10 Report
PLAT199_ALERT_1_G Reported _cell_measurement_temperature ..... (K)       293 Check
PLAT200_ALERT_1_G Reported   _diffrn_ambient_temperature ..... (K)       293 Check
PLAT791_ALERT_4_G The Model has Chirality at C10     (Chiral SPGR)         R Verify
PLAT791_ALERT_4_G The Model has Chirality at C11     (Chiral SPGR)         R Verify
PLAT791_ALERT_4_G The Model has Chirality at C22     (Chiral SPGR)         S Verify
PLAT791_ALERT_4_G The Model has Chirality at C23     (Chiral SPGR)         S Verify
PLAT899_ALERT_4_G SHELXL97   is Deprecated and Succeeded by SHELXL       2014 Note
```

```
   1 ALERT level A = Most likely a serious problem - resolve or explain
   1 ALERT level B = A potentially serious problem, consider carefully
  10 ALERT level C = Check. Ensure it is not caused by an omission or oversight
   9 ALERT level G = General information/check it is not something unexpected

   2 ALERT type 1 CIF construction/syntax error, inconsistent or missing data
   7 ALERT type 2 Indicator that the structure model may be wrong or deficient
   5 ALERT type 3 Indicator that the structure quality may be low
   5 ALERT type 4 Improvement, methodology, query or suggestion
   2 ALERT type 5 Informative message, check
```

It is advisable to attempt to resolve as many as possible of the alerts in all categories. Often the minor alerts point to easily fixed oversights, errors and omissions in your CIF or refinement strategy, so attention to these fine details can be worthwhile. In order to resolve some of the more serious problems it may be necessary to carry out additional measurements or structure refinements. However, the purpose of your study may justify the reported deviations and the more serious of these should normally be commented upon in the discussion or experimental section of a paper or in the "special_details" fields of the CIF. checkCIF was carefully designed to identify outliers and unusual parameters, but every test has its limitations and alerts that are not important in a particular case may appear. Conversely, the absence of alerts does not guarantee there are no aspects of the results needing attention. It is up to the individual to critically assess their own results and, if necessary, seek expert advice.

Publication of your CIF in IUCr journals

A basic structural check has been run on your CIF. These basic checks will be run on all CIFs submitted for publication in IUCr journals (*Acta Crystallographica*, *Journal of Applied Crystallography*, *Journal of Synchrotron Radiation*); however, if you intend to submit to *Acta Crystallographica Section C* or *E* or *IUCrData*, you should make sure that full publication checks are run on the final version of your CIF prior to submission.

Publication of your CIF in other journals

Please refer to the *Notes for Authors* of the relevant journal for any special instructions relating to CIF submission.

Validation response form

Please find below a validation response form (VRF) that can be filled in and pasted into your CIF.

```
# start Validation Reply Form
_vrf_PLAT089_preex3a
;
PROBLEM: Poor Data / Parameter Ratio (Zmax < 18) ........       6.52 Note
RESPONSE: ...
;
_vrf_PLAT303_preex3a
;
PROBLEM: Full Occupancy H-Atom  H2A    with # Connections       2.00 Check
RESPONSE: ...
;
_vrf_PLAT340_preex3a
;
PROBLEM: Low Bond Precision on  C-C Bonds ...............    0.00525 Ang.
RESPONSE: ...
;
# end Validation Reply Form
```

PLATON version of 24/11/2016; check.def file version of 23/11/2016

Datablock preex3a - ellipsoid plot

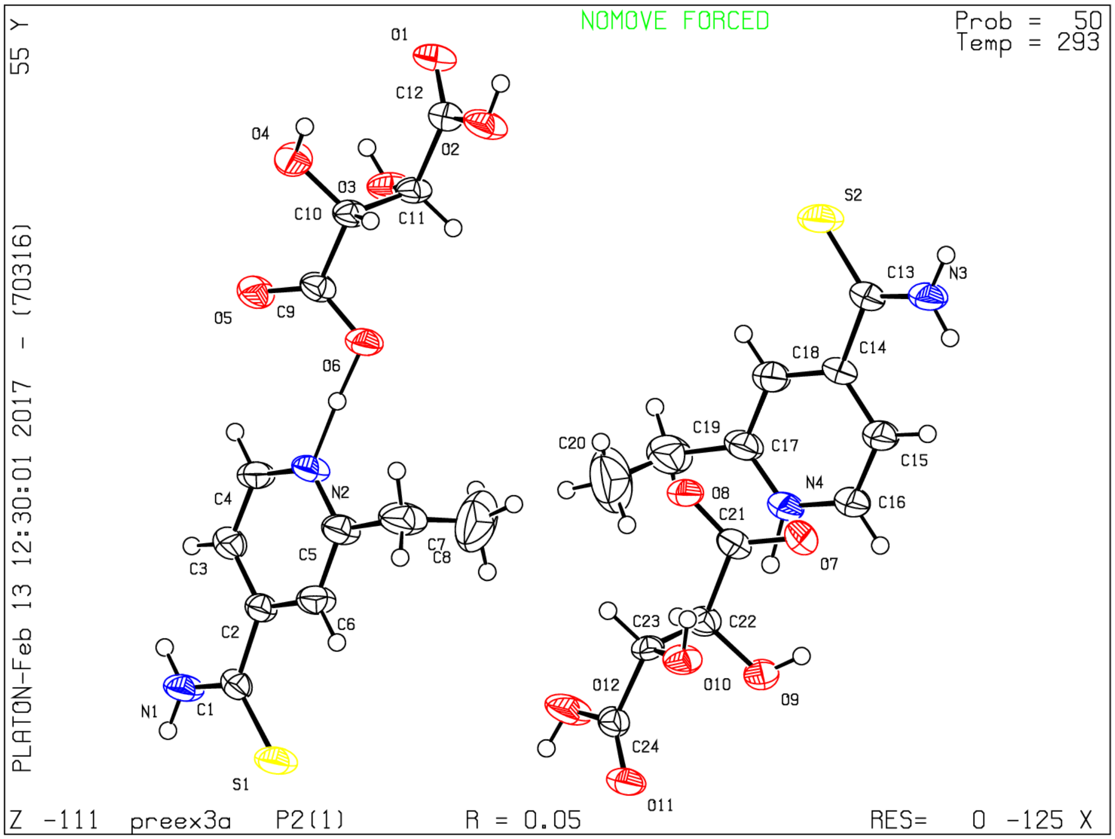

Supplement: Supplementary file 1 [file checkcif ETN-TAR.pdf › pharmaceutics-900610-Supplementary/checkcif ETN-TAR.pdf]
